# Quantitative comparison of ABC membrane protein type I exporter structures in a standardized way

**DOI:** 10.1016/j.csbj.2018.10.008

**Published:** 2018-10-18

**Authors:** Georgina Csizmadia, Bianka Farkas, Zoltán Spagina, Hedvig Tordai, Tamás Hegedűs

**Affiliations:** a MTA-SE Molecular Biophysics Research Group, Hungarian Academy of Sciences, Budapest, Hungary; b Department of Biophysics and Radiation Biology, Semmelweis University, Budapest, Hungary; c Faculty of Information Technology, Pázmány Péter Catholic University, Budapest, Hungary

**Keywords:** ABC proteins, Membrane proteins, Structure comparison, Structure validation, Quantitative structural properties, ABC, ATP binding cassette, CFTR, cystic fibrosis transmembrane conductance regulator, CG, coarse grained, CH, coupling helix, COG, center of geometry, ICD, intracellular domain, NBD, nucleotide binding domain, TH, transmembrane helix, TM, transmembrane, TMD, transmembrane domain

## Abstract

An increasing number of ABC membrane protein structures are determined by cryo-electron microscopy and X-ray crystallography, consequently identifying differences between their conformations has become an arising issue. Therefore, we propose to define standardized measures for ABC Type I exporter structure characterization. We set conformational vectors, conftors, which describe the relative orientation of domains and can highlight structural differences. In addition, continuum electrostatics calculations were performed to characterize the energetics of membrane insertion illuminating functionally crucial regions. In summary, the proposed metrics contribute to deeper understanding of ABC membrane proteins' structural features, structure validation, and analysis of movements observed in a molecular dynamics trajectory. Moreover, the concept of standardized metrics can be applied not only to ABC membrane protein structures (http://conftors.hegelab.org).

## Graphical abstract

**Figure:**
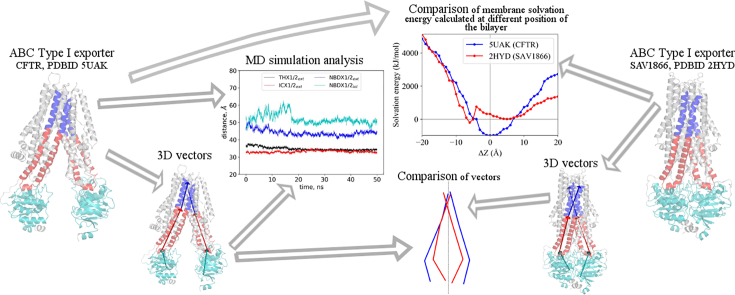

## 1 Introduction

ABC membrane proteins play important roles in many physiological processes from bacteria to man. They translocate substrates through the membrane bilayer or regulate channel function involving ATP binding and hydrolysis [[1], [2], [3]]. The functional expression of ABC membrane proteins can be altered either by mutations or regulatory processes [2], leading to various pathological states. The most known disorder caused by an ABC membrane protein is cystic fibrosis. Several mutations in the CFTR (cystic fibrosis transmembrane conductance regulator; ABCC7) chloride channel cause cystic fibrosis, a monogenic disease with high morbidity and mortality [[4], [5], [6]]. Most of the mutations affect protein folding and stability [7]. A decrease in the functional expression of the CFTR channel leads to a reduced chloride conductance in the epithelia resulting in imbalanced salt and water homeostasis. Although high throughput screening efforts resulted in molecules rescuing some CFTR variants (e.g. the G551D mutant), none of the identified drugs sufficiently restores the functional expression of ΔF508, the most frequent CFTR mutant [[8], [9], [10]]. The lack of high-resolution structural information has hindered drug development [11,12]. Because of cystic fibrosis and other ABC protein related disorders, understanding the relations between the conformations and function of ABC membrane proteins is of great importance. An increasing number of ABC exporter structures have been solved recently, which can be divided into two groups [13,14]. There are a higher number of Type I (P-glycoprotein-like) structures determined, which exhibit two large transmembrane domains, usually each built from six helices and two cytosolic nucleotide binding domains (Fig. S1). Their specific feature compared to ABCG-like transporters is the so called intracellular loops or domains, which are the continuation of TM helices in the cytosol. These “loops” contain coupling helices interacting with the nucleotide binding domains [15]. Structures in the absence of ATP exhibit highly separated NBDs or NBDs with contacts only at the opposite site of coupling helix interactions. In this apo conformation the TM domains expose a cavity towards the cytoplasm [14]. Therefore, these conformations are called “bottom-open, inward-facing” and “bottom-closed, inward-facing”, respectively. Upon binding of two ATP molecules, NBDs form a tight interaction and rearrange the transmembrane helices to close the cavity at the cytosolic side and open it to the extracellular space. Structures without opening at the extracellular region are also observed and suggested to form an intermediate of the transport cycle [16,17]. These conformations are named “bottom-closed, outward-facing” and “occluded” (bottom- and top-closed), accordingly.

Although all of the ABC membrane protein structures contain valuable information, there are several debates in the field. The “bottom-open, inward-facing” conformations are questioned based on the constant-contact model of the mechanism of function [18]. In addition, the wide separation of NBDs was not stable in molecular dynamics simulations and the large distance may be contributed to crystal contacts [14,19]. Similarly, the largely top-opened “bottom-closed, outward-facing” structures have also been criticized and suggested to be formed by the lack of the lateral pressure originating from the membrane bilayer during crystallization [20]. It is also challenging to interpret the unusual break of a transmembrane helix in the ABCC7/CFTR chloride channel structures determined by Chen et al. [[21], [22], [23]], since it is not present in other CFTR structures (R. Ford et al. unpublished and [24]) or other transporters of the same subfamily.

Despite the large efforts of transmembrane structure determination in the last decades, ABC membrane proteins, as well as membrane proteins in general are significantly underrepresented in the PDB database compared to globular proteins (http://pdbtm.enzim.hu, http://blanco.biomol.uci.edu/mpstruc). Even the revolution in cryo-electron microscopy (EM) methods [25,26] did not significantly decrease this difference in the last years. Moreover, diffracting crystals and cryo-EM images of membrane proteins exhibit low resolution confining their structure determination [27]. Therefore structure validation and assessing the quality of membrane protein structures are crucial not only in the field of ABC proteins but for all types of membrane proteins. For structure curation and validation the wwPDB has launched a tool, OneDep, based on recommendations of experts in crystallography, NMR, and cryo-EM [28]. These metrics (e.g. clashes between atoms, Wilson B-value [29,30], and the fit between R and R_free_ [31]) are extremely important and are the basis for a high quality database of curated 3D structures. However, they cannot provide higher-level information on the validity or possible distortion of domain-domain orientation caused by experimental conditions. Higher level comparison of protein structures have been initiated and organized in the CoDNaS database, but this tool exposes the limitation of being able to compare the structures of the same protein [32]. A comparative molecular dynamics (MD) study has been recently performed to assess various conformations of ABCB1/MDR1 [33], but the application of MD for a higher number of structures are highly resource intensive.

In order to address issues associated with the increasing number of ABC membrane protein structures and conformations, we aim to define metrics that can characterize structural properties at a higher level. We demonstrate that various vectors defined based on specific structural features of a protein family can highlight specific differences in conformations and alterations in structures. In addition, membrane solvation energy calculations can draw attention to functionally important regions.

## 2 Methods

### 2.1 ABC Membrane Protein Structures

Coordinates of ABC Type I exporters and information on membrane orientations were downloaded from the OPM database or from the PDB followed by the calculation of membrane region using the PPM server (Table S1) [34,35]. The structure determination method was collected from the PDB files, while the conformational state was determined by visual inspection. Transmembrane helix boundaries were extracted from OPM and PPM. Other regions, such as coupling helices, were identified semi-manually. PyMOL was used for structure and surface electrostatics visualization (The PyMOL Molecular Graphics System, Version 1.8.4 Schrödinger, LLC). The orientation of the structures was standardized by the rotation and translation of structures to a selected reference structure. As a reference we selected a structure of TM287/288 (PDBID: 3QF4), which exhibits intermediate 3D properties, such as inward-facing TMDs and bottom-closed NBDs [36].

### 2.2 Comparison of Transmembrane Region Localizations

Selected resources include OPM [34,35], PDBTM [37,38], and MEMPROTMD [39] databases. The positioning of the structures in the membrane bilayer was characterized by the tilting angle and the localization along the Z axis. We compared the values from each set to the values from the OPM as a reference. The protein tilting was calculated by the angle between the membrane normal and the principal axis of the protein, which axis is set by the bisector between the THX1 and THX2 conftors (CONFormational vecTORs; TH4–5 and TH10–11). In the case of the CG structures the membrane normal was calculated by subtracting the center of geometry of PO4 and NC3 beads in the upper leaflet from that in the lower leaflet. The vector normal of the plane defined by three DUMMY atoms were used for OPM entries. The difference in the membrane centers was determined after structural alignment. Regarding the CG structures, the back-bone beads were aligned to the Cα atoms of the all-atom structures. The reference membrane center was calculated as the center of geometry (COG) of the DUMMY membrane atoms from OPM. The membrane center of systems from CG simulations was determined by the COG of the PO4 and NC3 beads.

### 2.3 Calculation of Helix Properties and Vectors

The bending, rotation, and twist of helices were calculated using MDAnalysis and HELANAL [40,41]. Conftors were calculated using Python scripts combining the numpy and MDAnalysis packages (Table S2). To provide a simplified and visual comparison of structures, conftors were plotted using Python Matplotlib.

### 2.4 Molecular Dynamics Simulations

Coarse-grained simulations were performed with structures of PDB entries 2HYD, 3Qf4, 4KSB, 5UAK, 5TSI, 5UJ9, and 4PL0 using GROMACS with the MARTINI force field (elnedyn22) [42,43]. Trajectories of all-atom simulations have been obtained using GROMACS as described earlier [44]. More details on simulation parameters are in the supplementary material.

### 2.5 Electrostatics Calculations

PDB2PQR [45] was run with PARSE force field [46], pH 7.0 and the option to create input template. Structural preparation needed for low resolution structures was made by VMD's Automatic PSF Builder. The input template for APBS [47] was modified to add 150 mM Na^+^ and Cl^−^ ions with charge 1 and −1, and radius 0.95 and 1.81 Å, respectively. For membrane solvation calculations APBSmem [48] was run with the following parameters (Table S4): Grid dimensions and fine grid size for x and y coordinates was collected from the input file of APBS. The z coordinate of the fine grid size was −2*z_min_ + 40 Å, where z_min_ is the smallest z coordinate from the PQR file. This way the whole protein was included in the fine grid, even when membrane was moved with ±20 Å. Medium grid size was 2 times and coarse grid size was 5 times of the fine grid size. Grid dimension was 161 for each axis. The membrane thicknesses from OPM were used and the flooding algorithm of APBSmem was used as the membrane filling method.

## 3 Results

### 3.1 Positioning of ABC Type I Exporters in Membrane Bilayers

The tilting of the protein relative to the bilayer normal and the location of the hydrophobic bilayer core are important features of protein conformation. The experimental information on tilting and insertion at the atomic resolution is highly limited, thus we assessed the membrane orientation of ABC membrane proteins using various computational methods. We compared data on membrane bilayer boundaries from OPM [34,35], PDBTM [37,38], and MEMPROTMD [39] databases. OPM calculates and minimizes the transfer free energy of transmembrane proteins at different values of distance from the bilayer center, bilayer thickness, and tilting. PDBTM's TMDET algorithm is a geometrical approach utilizing an objective function dependent on amino acid hydrophobicity. In the MEMPROTMD database membrane protein structures in a bilayer are generated using coarse grain simulations. Since we found ABC membrane proteins with large conformational changes in MEMPROTMD (e.g. transition from the bottom-open to the bottom-closed conformation), which changes may influence the interactions with lipid molecules, we performed CG simulations using MARTINI [43,49] on a selected set of ABC membrane protein structures as well.

**Fig. 1:**
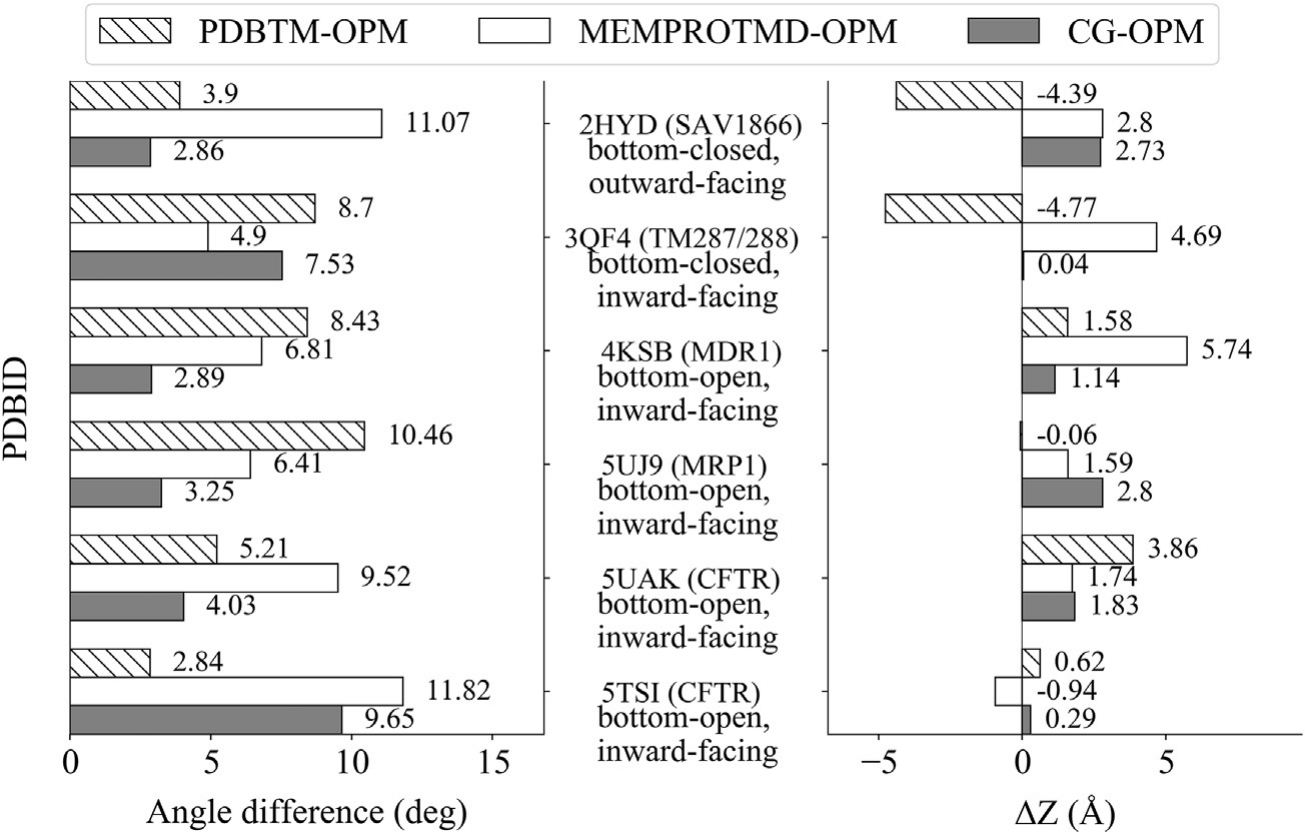
**Comparison of bilayer location around ABC membrane proteins by in silico methods.** Differences in the tilting angle of proteins in the membrane and the z-positioning of the membrane bilayer around proteins are depicted for selected ABC Type I exporter structures. Values extracted from PDBTM, MEMPROTMD, and our CG simulations are compared to values from OPM. OWF: outward-facing, IWF: inward-facing. See also Fig. S2.

We extracted the tilt angle and the location of the bilayer around each ABC membrane protein structure (Fig. 1, Fig. S2, and Table S1). The tilting was calculated as the angle between the membrane normal and the principal axis of the protein. This axis is set by the bisector between the vectors defined by two pairs of TM helices (TH4–5 and TH10–11), which cross-over to the opposite NBD, discussed below as THX1 and THX2 conftors (CONFormational vecTORS).

In most of the cases the difference in tilting obtained from different databases is negligible (below 6°). The largest differences were 10–12°, which seem significant by visual inspection, but correspond to a difference in immersion by only a few amino acids (1–2 helical turns) for the helices most distant from the central z-axis.

**Fig. 2:**
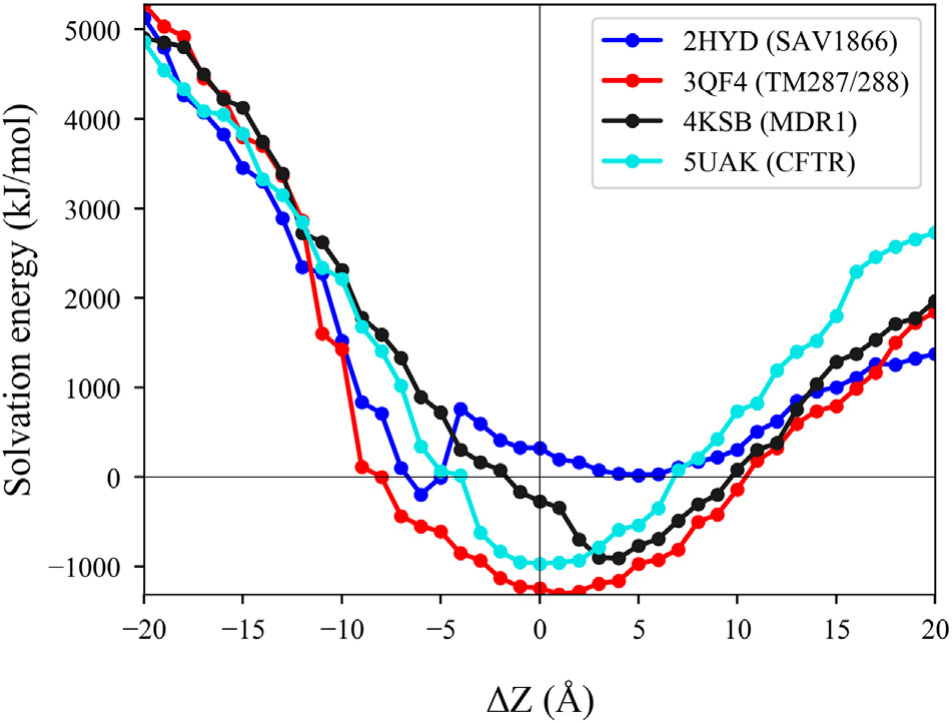
**Orientation in membranes assessed by APBS calculations.** Membrane solvation energy was calculated using APBSmem and shown for selected ABC membrane proteins. Calculations were performed for each protein at different positions of the bilayer (1 Å steps). Zero point is the location of the bilayer defined by OPM.

The bilayer location around the protein was characterized by the distance along the z-axis between the COG of transmembrane helices based on OPM annotation and the bilayer center. Most of the differences were negligible in z-location, except for a few structures, including 2HYD, 3QF4, 4KSB, and 5UAK. Therefore, we assessed the membrane embedment of these structures by membrane solvation calculation using APBS (Fig. 2).

Membrane solvation energy of 3QF4 has a minimum at the 0 distance, which means an agreement with OPM. Membrane insertion predictions of 4KSB by APBS agree with that of MEMPROTMD. However, the membrane insertion for 2HYD derived from APBS calculations is similar to that from PDBTM.

We conclude that the OPM, PDBTM, MEMPROTMD, and our CG simulations exhibit only slight differences in the investigated measures and it cannot be ascertained which method provides a prediction correlating the best with in vivo conditions. In this study we used the OPM database, since it exhibited the less deviation from the other methods.

### 3.2 Helix Bending and End-Locations Facilitate Structure Comparison and Highlight Critical Differences Between Conformations

In order to understand distinctive features of various ABC Type I exporter conformations and select critical 3D properties for the description of structures, we characterized various geometric properties of TM helices. We calculated the bending, the rotation, and the twist of helices [40] (TH2–5 and TH8–11), which have longer intracellular parts interacting with NBDs for each conformation class. In the “occluded” class there are positions with higher values of bending angles (Fig. S3). The inspection of these conformations revealed that there was a particular structure (T1SS, Type-1 secretion system, PDBID: 5L22) with high bending angle values originating from breaks in several TM helices.

**Fig. 3:**
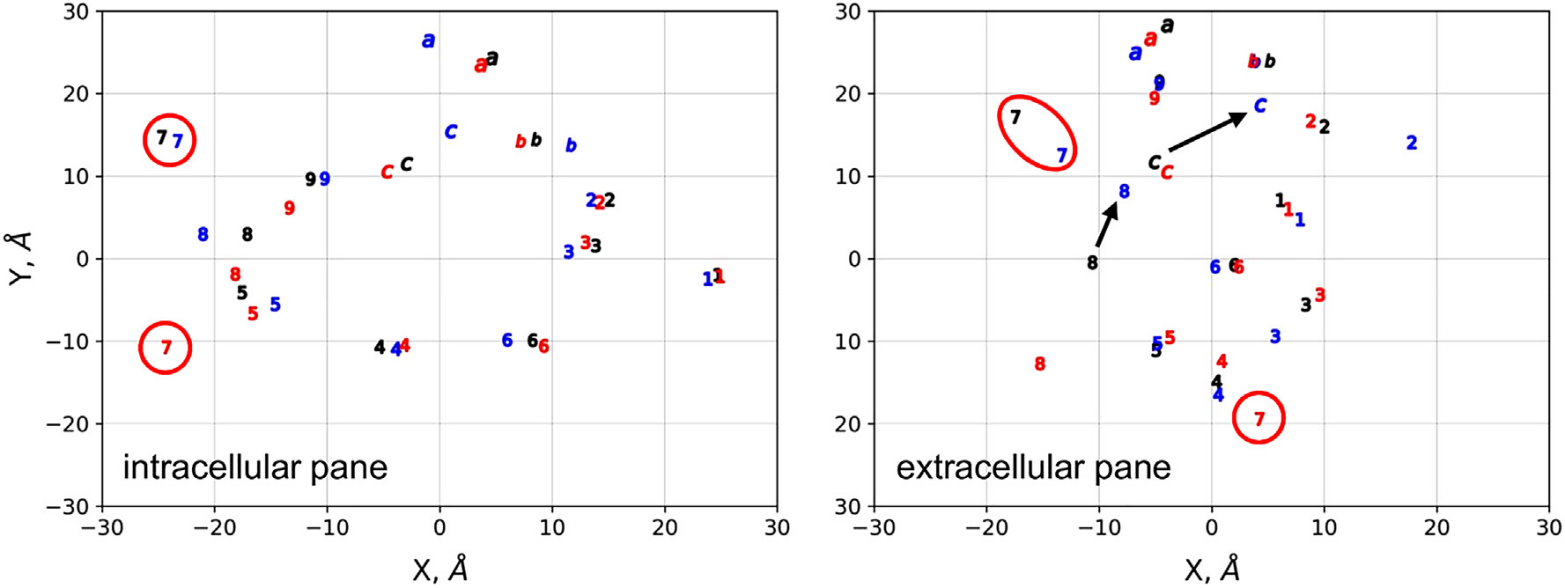
**The distance between the ends of TM helices highlight differences in conformations.** The intracellular (left) and extracellular (right) ends of TM helices in apo (PDBID: 5UAK, black; Fay et al., red) and ATP-bound (PDBID: 5W81, blue) CFTR structures were projected into 2D. The unusual localization of TH7 is highlighted by red circles. Arrows indicate the altered extracellular position of TH8 and TH12 in the ATP-bound structure. See also Fig. S4.

In order to visualize the relative orientation of TM helices, their intracellular and extracellular end positions were projected into 2D (Fig. S4). The application of these projections is demonstrated on the CFTR (ABCC7) cryo-EM structures [[21], [22], [23], [24]], which have been received an intense attention. The projections in Fig. 3 reveal that TH7 in the structure of Fay et al. [24] is located at a completely different position compared to other structures [[21], [22], [23]] (and also to an unpublished electron density map by Ford et al., University of Manchester, UK). The relocated TH7 and TH8 are claimed to be a result of the ~200 a.a. disordered regulatory domain connected to the N-terminus of TH7. Fig. 3 also reveals that there are only small differences in the relative localization of the intracellular ends of TM helices between the apo and ATP-bound conformations, while extracellular ends of TH8 and TH12 are repositioned in the ATP-bound structure (Fig. 3, arrows). The comparison of these two structures reveals the closure of the NBDs and the associated dislocation of some intracellular helices, while the TM helices are practically unchanged, except the above mentioned two extracellular ends.

### 3.3 Defining Conftors Sensitive to Overall and relative Domain Conformations

**Fig. 4:**
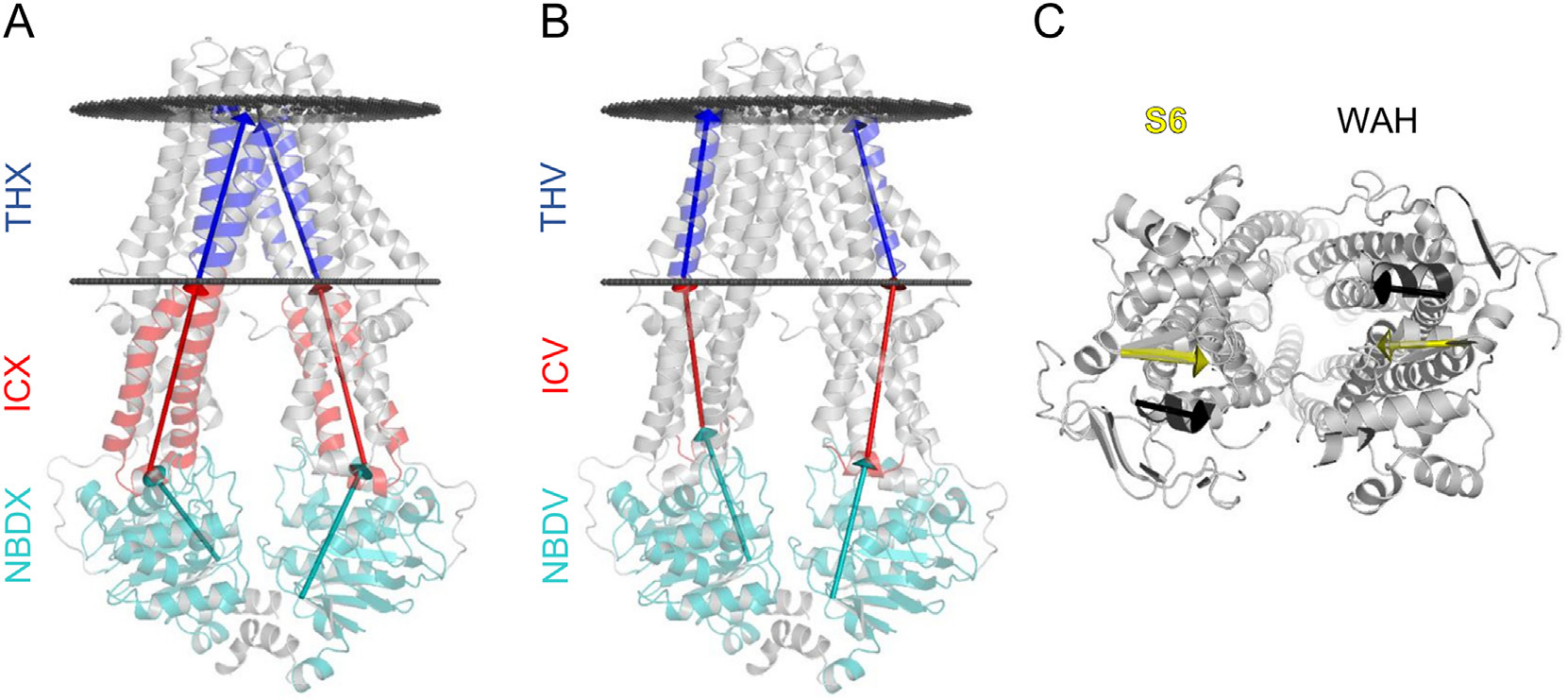
**ABC conftors: dedicated vectors to describe ABCType I exporter conformation. (a, b)** Vectors are defined by either a single Cα or the center of mass of more Cα. Conftors in the membrane region point from the intracellular to the extracellular ends of helices (blue; a: THX1 and THX2, b: THV1 and THV2). Conftors for the intracellular domains (a: ICX1 and ICX2, b: ICV1 and ICV2) and nucleotide binding domains (a: NBDX1 and NBDX2, b: NBDV1 and NBDV2) are red and teal, respectively. **(c)** Conftors are also defined between the Walker A helices (black) and strand S6 (yellow with black line) of the opposite NBDs.

We clustered ABC membrane protein structures on pairwise RMSD values. However, because of the composite nature of RMSD, some members of a cluster also exhibit crucial structural variations compared to other members (Fig. S5). Therefore, we aimed to characterize conformations using vectors defined in a manner to pin differences in intra- and inter-domain arrangements (Fig. 4).

We propose that simplified representation of protein conformations using conftors (CONFormational vecTORS), carefully selected standardized vectors based on high resolution structures help to interpret differences between protein structures. As a proof of principle, we demonstrate the definition and application of conftors in the case of ABC transmembrane proteins.

Since the function of ABC membrane proteins is coupled to conformational changes in the TM domains, we selected vectors that are capable to describe differences in the orientation of transmembrane helices. For example, to compare the level of opening towards the extracellular space (e.g. the conformation of the outward-open 2HYD and the outward-closed, occluded conformation of 4PL0, Fig. S6) we defined the THV1–2, THX1–2 and THC3–9 conftors (Table S2). As the orientation and bending of an individual α-helix may be deviated in a specific structure, defining the ends of TMD conftors as the COG of Cα at the ends of two α-helices may be justified in some cases (THX, Fig. 4a). These THX conftors characterize the conformation of the transmembrane helices, which cross over from one TMD to the opposite NBD. While the angle between THV1 and THV2 conftors can separate only the bottom-closed, top-closed conformations with an average value of 23° compared to all other conformations with values between 36 and 40°, angle enclosed by the THX conftors is able to make distinction between “bottom-open, inward-facing” (46°), “bottom-closed, top-closed” (26°), and the two other conformations (39° and 35°) (Table S3).

Since the so-called intracellular loops or domains (ICDs), which are the continuation of TM helices, can enclose an angle with the membrane-embedded parts of the TM helices, we set separate conftors, ICV1–2 and ICX1–2 and for the transmembrane and intracellular parts of the TM helices (Fig. 4, Table S2). The angle between ICV1 and ICV2 differentiate the inward-facing (43° and 38°), the outward-facing (60°), and occluded (53°) conformations (Table S3).

The closed and open conformation of NBDs is usually determined easily by visual inspection. However, the extent of their separation and especially their orientation and rotation relative to each other and to the TMDs remain hidden. Therefore, we defined conftors NBDV1–2 and NBDX1–2 pointing from the COG of coupling helices to the first residue of S9, the last strand in NBD with small deviation among structures (Fig. 4a–b). NBDX conftors have slightly lower values for the inward-facing conformations compared to outward-facing or top-closed structures. The NBDX1/NBDX2_ext_ conftor's length (the distance of the NBD/TMD interface regions) reveals differences not only between inward-facing and outward-facing conformations, but also between “bottom-open, inward-facing” and “bottom-closed, inward-facing” conformations (Table S3). The S6 conftor defined by the opposite strands S6 and the WAH conftor based on the opposite α-helices incorporating the Walker A motif are anticipated to depict the orientation of NBDs relative to each other and also to the TMDs (Fig. 4c).

### 3.4 Application of Conftors

**Fig. 5:**
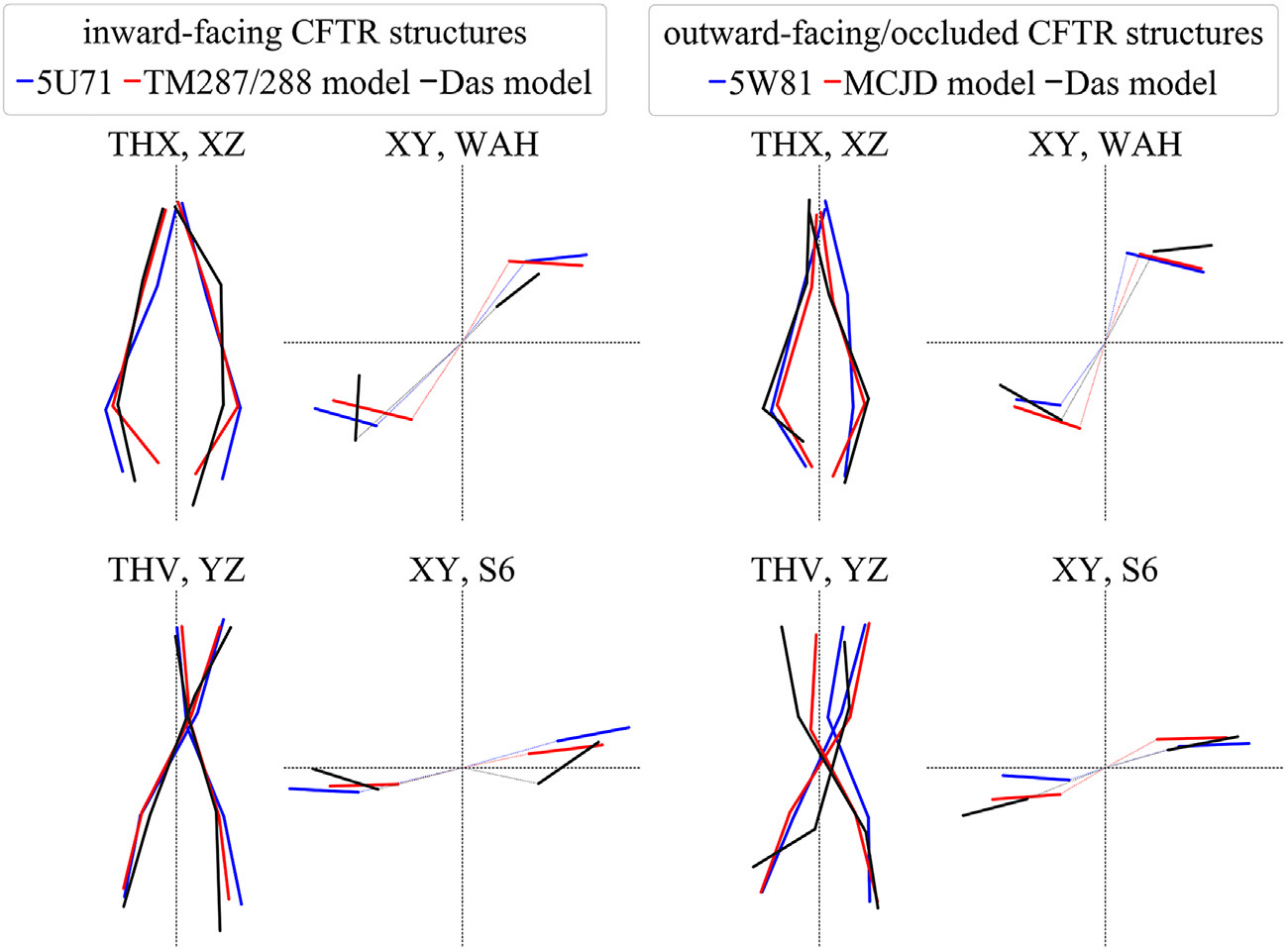
**Conftors highlight important similarities and differences among CFTR structural models.** The open Das model exhibits large deviations in NBD rotations indicated by WAH and S9 conftors. The closed Das model shows differences in both TM helix and NBD conformations when compared to other structural models, and the orientation of an NBD relative to the TMDs is not observed in any other ABC Type I exporter structure. CFTR cryo-EM structures are PDBIDs 5U71 and 5W81. TM287/288 and McjD based homology models have been generated by Corradi et al. CFTR models by Das et al. are from http://troll.med.unc.edu/cftr/. See also Fig. S6.

Conftors can also be used for visualization purposes. For example, the degree of opening is shown by the THC conftors (Fig. S6). A more exquisite example includes structural models of CFTR. The inward-facing apo cryo-EM structure exhibits properties similar to other inward-facing structures (PDBID: 5U71, Fig. 5). Since it does not exhibit an open pathway for chloride, a complex modeling complemented with experiments has been performed by Das et al. to generate a conformation with open channel [50]. This study is important, since even a phosphorylated and ATP bound CFTR structure (PDBID: 5W81) is not opened [23].

However, the conftors of these structures reveals that the models exhibit large deviations from known structures, which differences are hidden or attenuated in the 3D structure (Fig. 5). Their models show differences in the transmembrane regions, which can be anticipated because channel opening may require different conformational changes in the TM domains compared to active ABC transporters. In contrast, the differences in the intracellular domain orientations indicate inaccuracies.

**Fig. 6:**
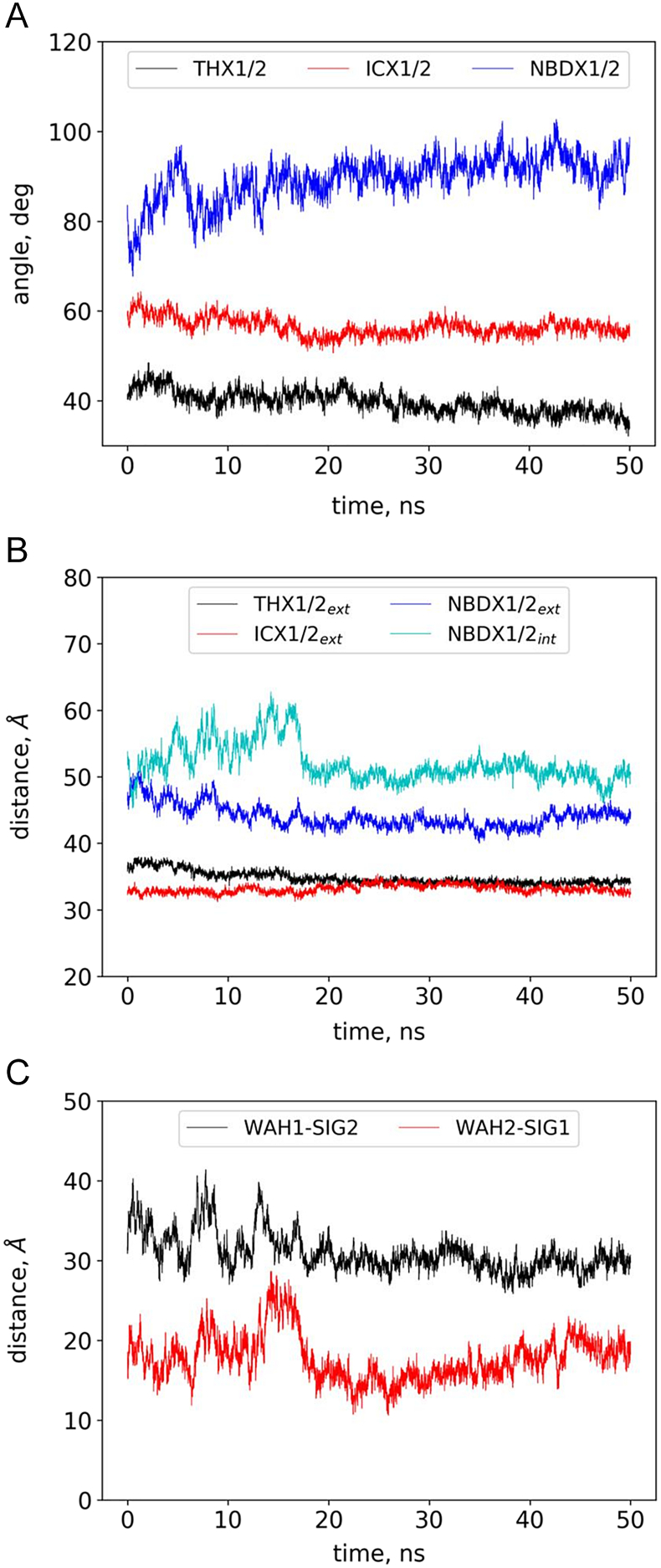
**Application of conftors for MD analysis.** Angles between conftors (**a**) and lengths of conftors (**b, c**) were calculated over the trajectory of an MD simulation with the CFTR bottom-open, inward-facing structure (PDBID: 5UAK). NBD1 and NBD2 got into contact at around 18 ns of the simulation.

Conftors can also be effectively used for analysis of trajectories from molecular dynamics simulations. We have recently investigated the dynamics of the inward-facing CFTR cryo-EM structure and noticed the closure of the nucleotide binding domains (Fig. 3a in [44]). As the measures we have calculated were not sufficient to fully understand the movements of the protein in detail, we analyzed the trajectories employing conftors. Several angles between various conftors were calculated over the trajectories and plotted in Fig. 6. The conformation of the membrane embedded parts of the TM helices did not change largely, albeit a small decrease in angles of THX1 and THX2 can be observed (Fig. 6a, black), that may arise from the lateral pressure of the lipid bilayer [20]. The increase in the NBDX1/2 angle indicates that the bottom of the NBDs (the opposite site of the TMD/NBD interface) gets closer to each other. This event is clearly shown by the length of the NBDX1/2_int_ “distance” conftor (Fig. 6b, cyan), as NBDX1/2_int_ highly fluctuates till ~18 ns, when it became stable around 50 Å. The conftors describing the distance between Walker A and Signature motifs (WAH1-SIG2 and WAH2-SIG1) indicate the higher separation of WAH1 and SIG2 in the nonfunctional degenerate ATP-binding site-1, when compared to that of the canonical site-2 (Fig. 6c). This observation indicates an asymmetry in the association of the two NBDs.

### 3.5 Electrostatics Calculations Highlight Structural Hot Spots

Measures representing physicochemical properties are also crucial for structure characterization. Protein surface electrostatics can be used to characterize the overall conformation of transmembrane domains. The positive-inside rule should manifest in most of the transmembrane proteins [51,52], thus in structural context the amino acids in the region of the inner membrane boundary are expected to build up a positively charged ring around the protein. This positive ring (“blue collar”) is present in many experimental structures, such as calcium ATPases [53]. A quantitative description of this ring is quite challenging, but calculating and analyzing surface electrostatics in individual cases can be informative to localize protein-protein interactions. At interaction sites the positive ring is expected to be ceased. Indeed, at the intramolecular interaction site of the L0/Lasso motive preceding the first transmembrane domain in ABCC proteins, such as ABCC1/MRP1 (Fig. S7) and ABCC7/CFTR [44] the positive ring breaks with a hydrophobic spot. The functionally important amphipathic α-helix of the L0/Lasso motif binds to this patch as observed in several cryo-EM structures.

**Fig. 7:**
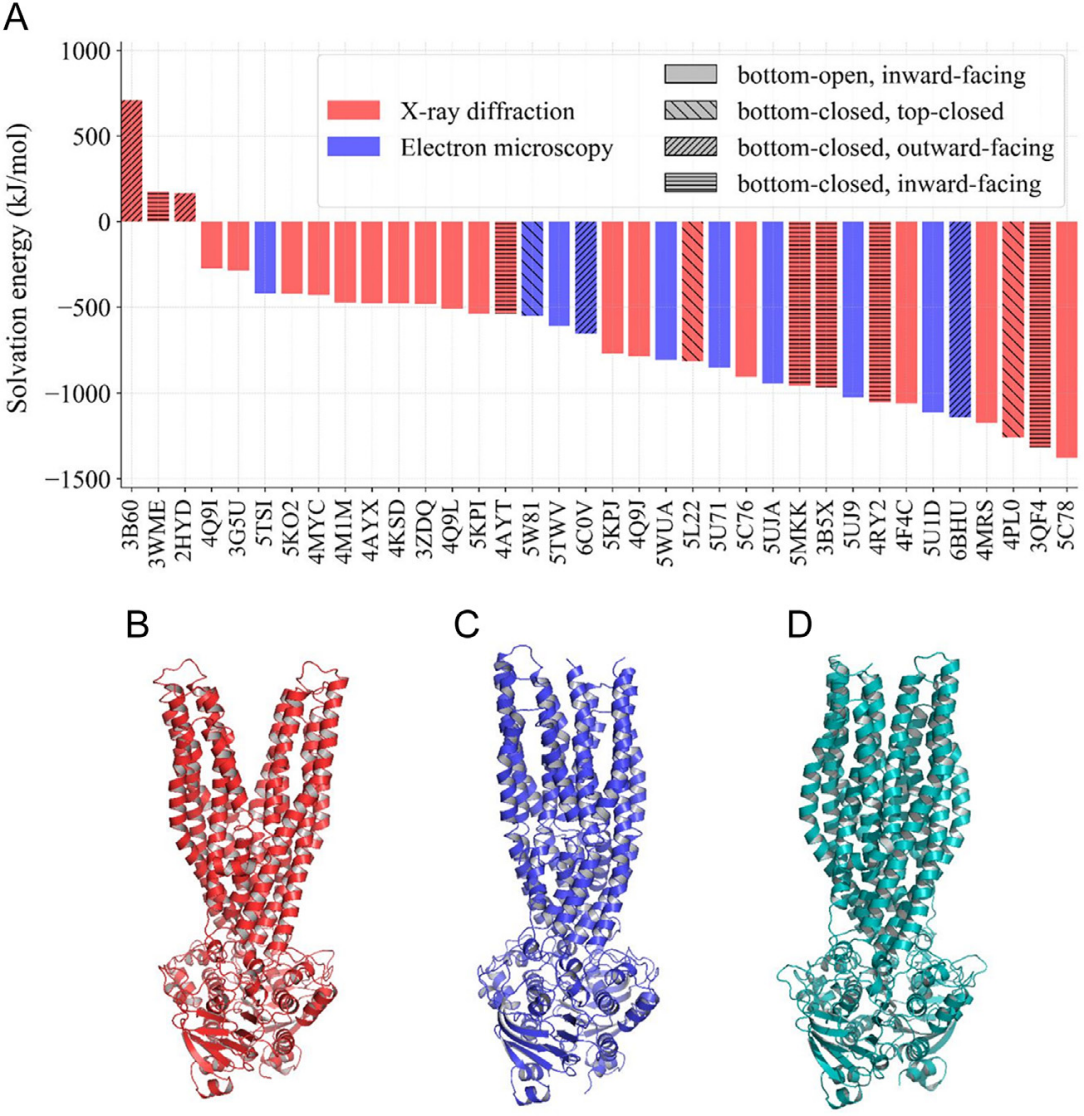
**Membrane solvation energy values are high for conformations with a large outward-facing cavity. (a)** APBSmem was used to calculate membrane solvation energy. Conformations with smaller (MRP1, PDBID: 6BHU and MDR1, PDBID: 6C0V) and larger (SAV1866, PDBID: 2HYD and MRP1, PDBID: 3B60) outward-facing cavities exhibit negative and positive solvation energies, respectively. Differences in the level of the opening are shown for Sav1866 (**b**) and MDR1/ABCB1(**c**). The wide opening of Sav1866 has been questioned and an alternative ATP-bound conformation has been proposed (Protein Model Database: PM0075213) (**d**).

APBS can also be applied for membrane solvation calculations. We computed the membrane solvation energy for each ABC Type I exporters and for each transmembrane helix from per amino acid contribution to the solvation, using APBSmem (Fig. 7). The total solvation energy spans from low negative to high positive values and we could not detect any correlation between the energy and some other property, such as determination method or resolution. Interestingly, the structures with the lowest and highest solvation energies were solved by X-ray. The two structures with the highest energy (SAV1866, PDBID: 2HYD and MsbA, PDBID: 3B60) are the two outward-open conformations suggesting that these widely open conformations may be caused by the lack of a bilayer under crystallization conditions. In contrast, the solvation energy of the outward-open MRP1 (PDBID: 6BHU) [54] and MDR1 (PDBID: 6C0V) [55] conformations, which are less open towards the extracellular space, is small.

## 4 Discussion

An increasing number of membrane protein structures are being determined. In the accompanying papers the new structures are compared to previously known ones and this comparison is usually semi-quantitative and not complete. In addition, while basic important metrics, which are general for all types of proteins (e.g. phi/psi angles, fit of the model to experimental data) are required to be calculated for validation, no quantitative and standardized measures have been defined to characterize geometric and physicochemical properties of structures. Importantly, earlier it was relatively straightforward which known conformations should be used for comparison to the new conformation because of the low number of available high-resolution structures. With the increasing number of solved structures, a set of standardized measures help to avoid a bias in reference structure selection and also in selecting structural regions for demonstrating novel and intriguing properties of the newly determined conformation. At this moment the low number of structures in certain membrane protein families limits the definition of conftors. For example, only one conformation has been determined for the ABCG2 and ABCG5/ABCG8 Type II exporters [13,56,57], therefore we could not test the usefulness of any conftor for the ABCG subfamily. Since in the case of ABC Type I exporters there are a larger number of “bottom-open” and “bottom-closed” conformations, numerous conftors could be defined and validated. Importantly, the existence of various conformations enabled us to evaluate vectors as conftors and discard which do not deliver information (e.g. THV conftors are not discriminative for the outward-facing and the inward-facing conformations, while THX conftors can differentiate these conformations well; Table S3).

Using various quantitative measures, we show how to demonstrate crucial differences between CFTR conformations for researchers other than structural biologists, since these differences, even in an ambiguously-defined form, have generated uncertainty in the field regarding the validity of the experimental structures. Actually, the slight differences between the apo and ATP-bound conformations, the membrane solvation of TH8 [44], and the dislocated TH7 and TH8 in the CFTR structure of Fay et al. [24] (Fig. 3) suggest that most likely the lipid environment (micelle) can have a profound effect on CFTR structure [58]. In the case of structures determined in a micelle, it is hard to imagine other factor than the lipid/detergent environment, playing a role in maintaining the conformation of the TM helices in the ATP-bound conformation highly similar to the apo conformation, while the intracellular parts of these helices (ICDs) and the NBDs exhibit a significant closure. On the other hand, the highly deviated TH7 and TH8 conformations of CFTR from different laboratories (Fig. 3) underscore the mobile nature of this region, which phenomenon has already been indicated by experiments. Most importantly, the above mentioned metrics can be useful not only for structure validation and comparison, but understanding the conformational changes associated to function (Fig. 3 and Fig. S4) and dynamics (Fig. 6). We emphasize that outliers of angles or membrane insertion energies may not indicate problems with a structure but may sign structurally or functionally important regions, as outlying phi/psi angles in the case of annexin [59].

The utilization of standardized metrics for structure validation and structure comparison aid the rigorous description of structural features and advance our knowledge on function-related conformations, thus help to understand the effect of mutations on protein structure and promote structure-based drug design. The proposed and similar metrics can be applied not only to the ABC membrane proteins. However, for other classes of proteins several vectors should be tested by an expert on the given protein family as long as no automatic algorithms are available. To overcome the difficulties of manual definitions of conftors, we are developing a web application and algorithms for generalized application of conftors (http://conftors.hegelab.org).

## Supplementary Data

Supplementary text and figures Image 1
