# Supplementary material for: Quantitative comparison of ABC membrane protein type I exporter structures in a standardized way
Source: Comput Struct Biotechnol J. 2018 Oct 18;16:396–403. doi: 10.1016/j.csbj.2018.10.008 (PMC6222291; doi:10.1016/j.csbj.2018.10.008)
Supplement: Supplementary file 1 — Supplementary text and figures [file mmc1.pdf]

Quantitative comparison of ABC protein structures in a standardized way

Georgina Csizmadia, Bianka Farkas, Zoltán Spagina, Hedvig Tordai, Tamás Hegedűs

SUPPLEMENTARY INFORMATION

Coarse grained MD simulations. *martinize.py* scripts was used to generate the CG protein structure [1]. The protein was solvated and inserted into a POPC bilayer using the *insane.py* script [2]. We employed the equilibration protocol of micelle builder of MARTINI/CHARMM-GUI [3], which algorithm employs a hole in the bilayer (generated *insane.py*, initial radius was set to 20 Å) making possible equilibration through flip-flop of lipid molecules by applying cylindrical repelling forces to the lipid tails. The hole is closed gradually with the following parameters: values of DPOSRES_FC, DVESICLE_LIPIDTAIL_R, integration step, and number of steps were (1000, 0.2, 0.02, 5 000 000), (1000, 0.15, 5 000 000), (500, 0.15, 0.01, 1 000 000), (200, 0.5, 0.015, 500 000), and (100, 0.2, 0.02, 100 000). The production run was unconstrained with an integration step of 0.02 and run for 100 000 000 step (2 μs). Other main MD parameters were set according to de Jong *et al.* [4]. The temperature and pressure are kept constant at 310 K and 1 bar, respectively.

All atom MD simulations. The input files for energy minimization, equilibration steps (NVT, NPT), and production run were generated at the CHARMM-GUI web interface [5]. The CFTR structure was oriented using the OPM (Orientations of Proteins in Membranes) database and inserted into homogenous POPC (1-palmitoyl-2-oleoyl-sn-glycero-3-phosphocholine) lipid bilayer. Simulations were forked from the equilibrated structure and consecutive sets of equilibration steps and production run. Berendsen thermostat and barostat were used in the equilibration steps, while Nose-Hoover thermostat and Parrinello-Rahman barostat with semiisotropic coupling were employed and LINCS algorithm was used to constrain bonds. Simulations were carried out in constant particle number, pressure, and temperature ensembles with a time step of 2 fs.

Figure S1. Structural organization of type I ABC exporters. (a, b) Two mMDR1 structures (PDIDs: 5KPJ, 3G5U) represents the “bottom-open, inward-facing” conformation. The two NBDs are open (far from each other) at different extents. **(c)** The TM287/288 structure (PDBID: 3QF4) possesses a “bottom-closed, inward-facing” conformation. **(d)** McjD (PDBID: 4PL0) exhibits a “bottom-closed, top-closed” (occluded) conformation, while **(e)** an MsbA structure (PDBID: 3B60) shows a “bottom-closed, outward-facing” conformation. TMD: transmembrane domain, ICD: intracellular domain or intracellular “loop”, NBD: nucleotide binding domain, CH: coupling helix.

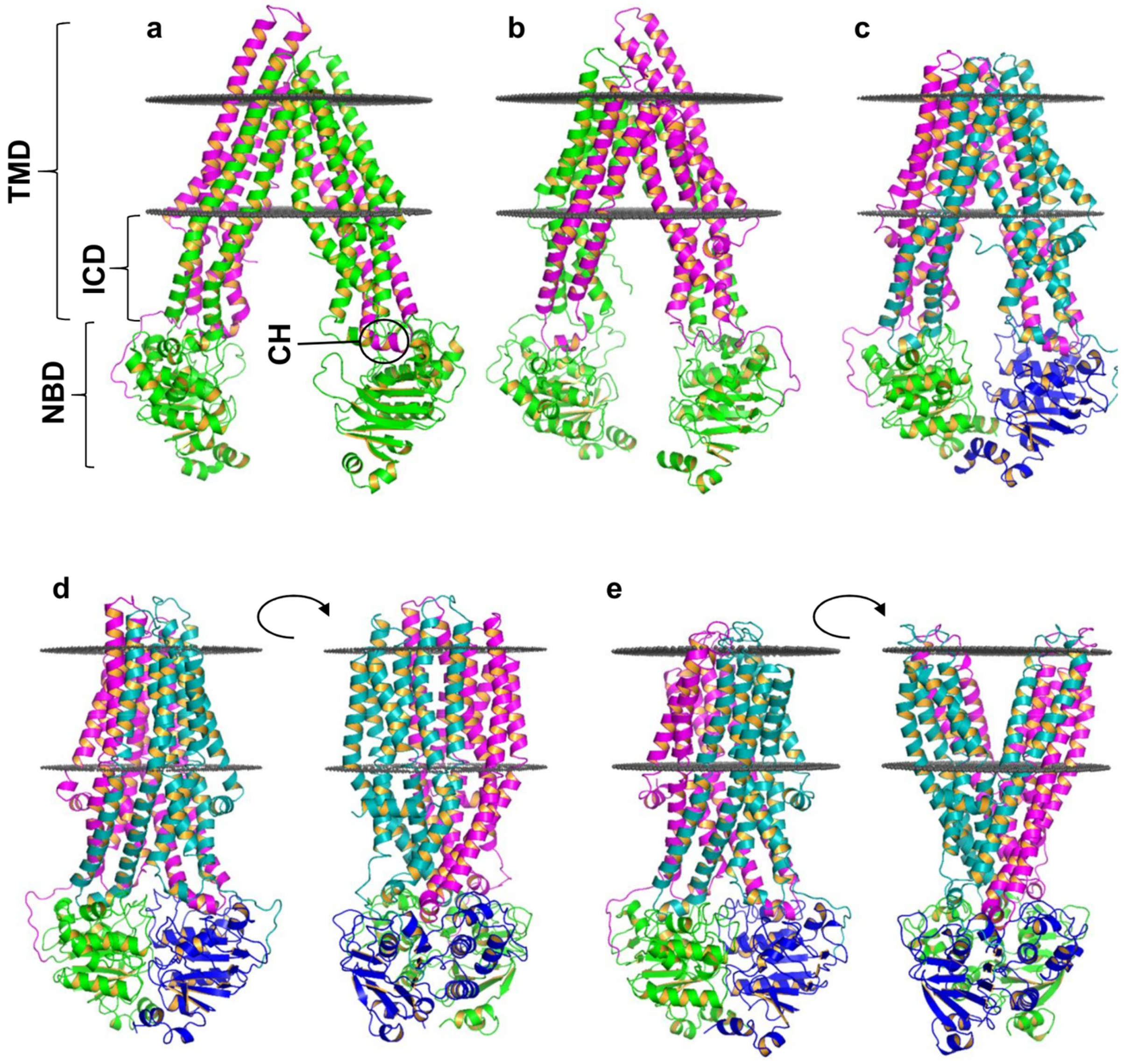

Figure S2. Comparing the bilayer location around ABC proteins by *in silico* methods. Differences in the tilting angle of ABC protein structures in the membrane and the predicted z-positioning of the membrane bilayer around the structures are shown. Values extracted from PDBTM, MEMPROTMD, and our CG simulations are compared to values from OPM. Only non-redundant structures are shown. Bilayer location for "bottom-closed, outward-facing", "occluded", and "bottom closed, inward-facing" structures (**a**) and for "inward-facing, bottom-open" structures (**b**) are shown.

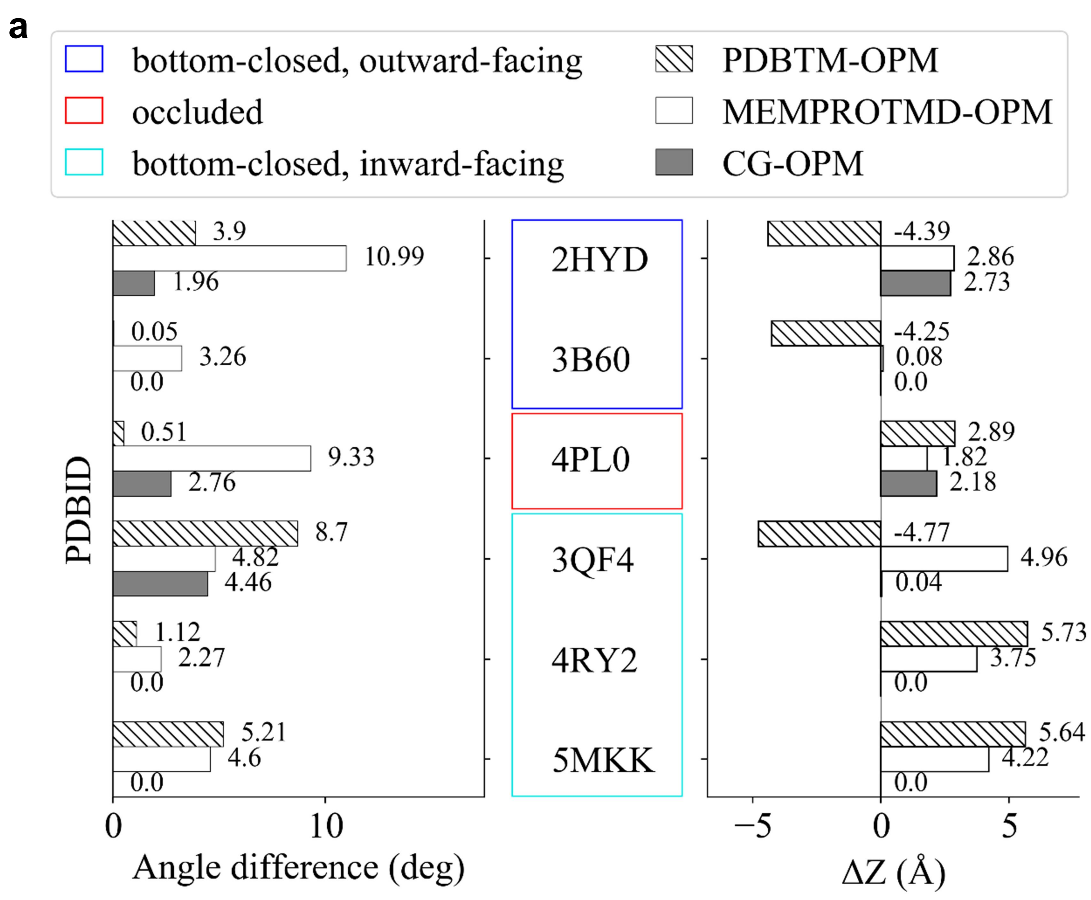

b

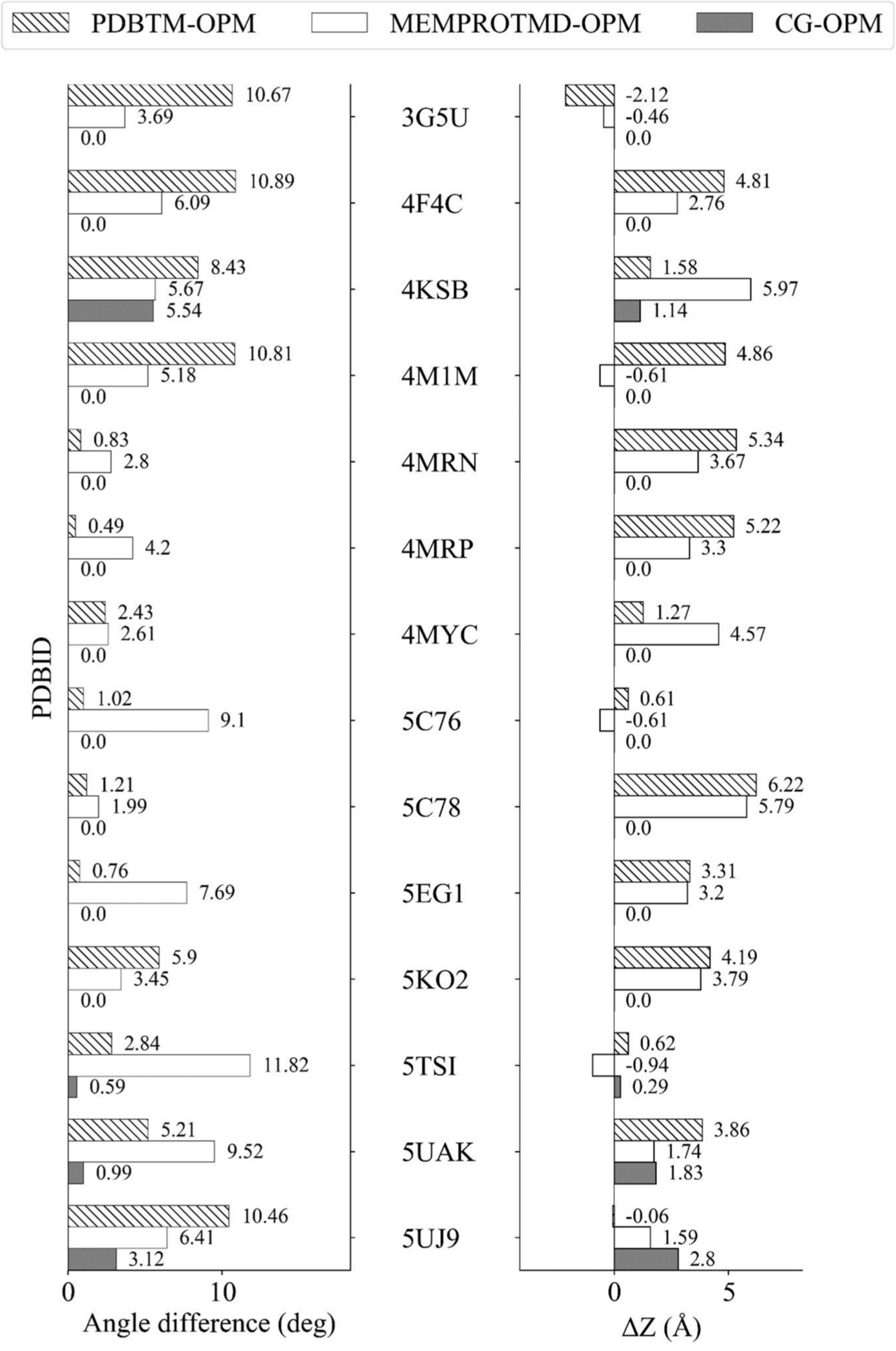
PDBTM-OPM
MEMPROTMD-OPM
CG-OPM
PDBID
3G5U
4F4C
4KSB
4M1M
4MRN
4MRP
4MYC
5C76
5C78
5EG1
5KO2
5TSI
5UAK
5UJ9
10.67
3.69
0.0
10.89
6.09
0.0
8.43
5.67
5.54
10.81
5.18
0.0
0.83
2.8
0.0
0.49
4.2
0.0
2.43
2.61
0.0
1.02
9.1
0.0
1.21
1.99
0.0
0.76
7.69
0.0
5.9
3.45
0.0
2.84
11.82
0.59
5.21
9.52
0.99
10.46
6.41
3.12
0
10
Angle difference (deg)
-2.12
-0.46
0.0
4.81
2.76
0.0
1.58
5.97
1.14
4.86
-0.61
0.0
5.34
3.67
0.0
5.22
3.3
0.0
1.27
4.57
0.0
0.61
-0.61
0.0
6.22
5.79
0.0
3.31
3.2
0.0
4.19
3.79
0.0
0.62
-0.94
0.29
3.86
1.74
1.83
-0.06
1.59
2.8
0
5
ΔZ (Å)

Figure S3. Bending of transmembrane helices. We calculated and averaged the bending of TM helices with intracellular domains for each conformational class. The values are aligned to the intracellular end of each TM helix (a.a. position 0). The larger angle values at the helix ends indicate the breaks at the coupling helices (in the negative range) and the extracellular loops (in the positive range). Several extreme values of bending angles can be observed in the case of the "occluded" ("bottom-closed, top-closed") conformations. For example, a detailed examination of these structures showed highly bended helices in T1SS (PDB: 5L22), which is shown below with broken helices in color (TH3 in chain A: blue, TH6 in chain A: magenta, TH3 in chain B: red, TH6 in chain B: yellow). It has been suggested that these breaks in the helices are required for the transport of very large, unfolded peptides and provide a different transport mechanism when compared to the alternating access mechanism of transporters of small substrates [6].

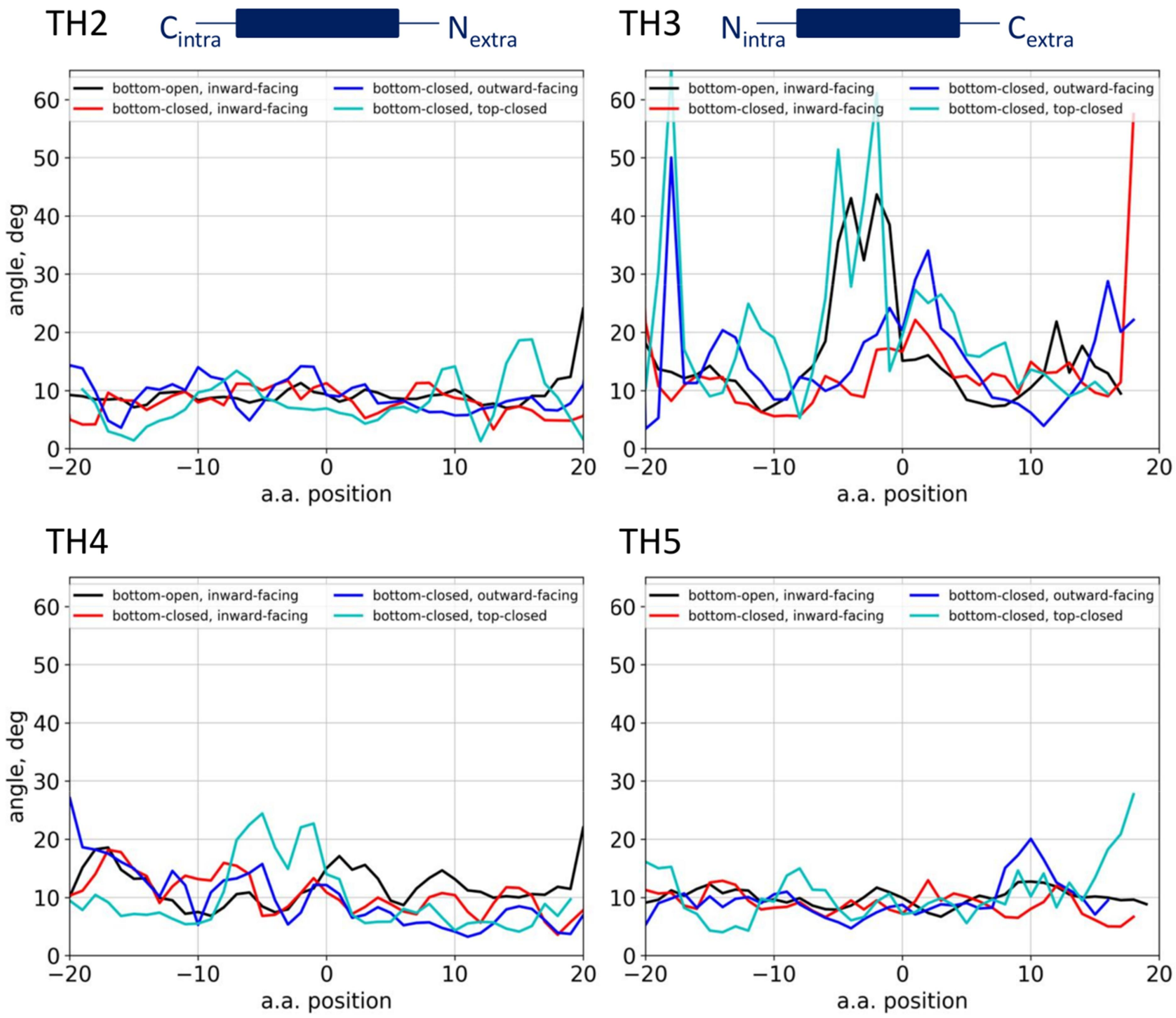

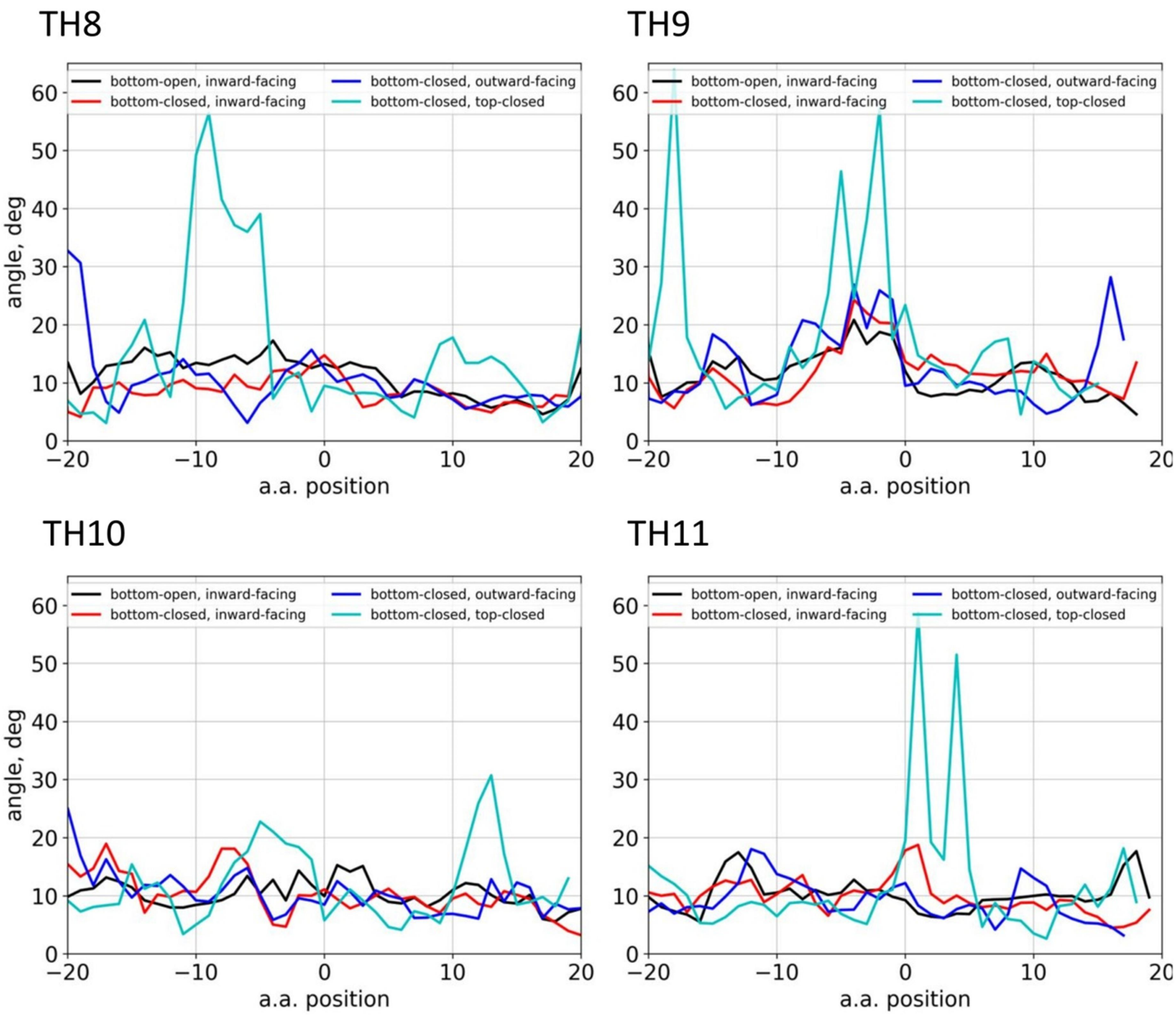
TH8
TH9
TH10
TH11
bottom-open, inward-facing
bottom-closed, inward-facing
bottom-closed, outward-facing
bottom-closed, top-closed
angle, deg
a.a. position
0
10
20
30
40
50
60
−20
−10
0
10
20

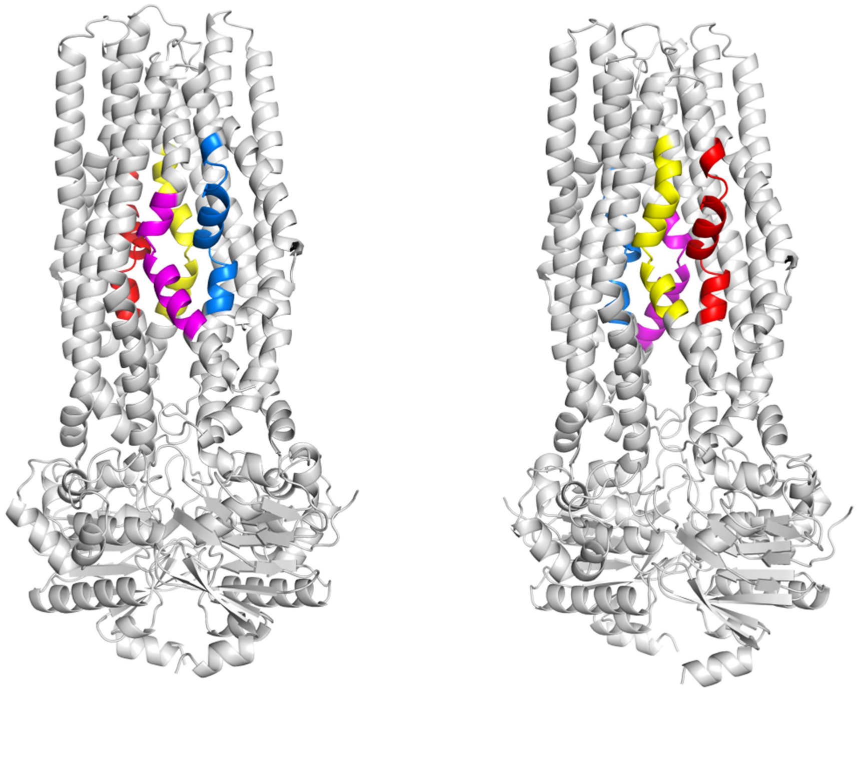

Figure S4. Projection of termini of transmembrane helices into 2D. The x and y coordinates of the Cα of the amino acid at the intracellular and extracellular ends of TM helices from ABC proteins are shown. TM helices 1-12 are labeled from ‘1’ to ‘9’ and ‘a’ to ‘c’. These plots can exhibit important differences between conformations. For example, the intracellular end of TH6 and TH12 does not get significantly closer to each other in the outward-facing conformations compared to that of the inward-facing conformations, but the intracellular end of TH3 and TH9 moves into the central cavity. While the extracellular ends of all of these helices get further from the central axis in the outward-facing conformations compared to that of the inward-facing conformations. This and similar observations help both in deeper understanding of differences between various functional conformations and in identification of conftors characteristic for a given conformation.

INTRACELLULAR ENDS

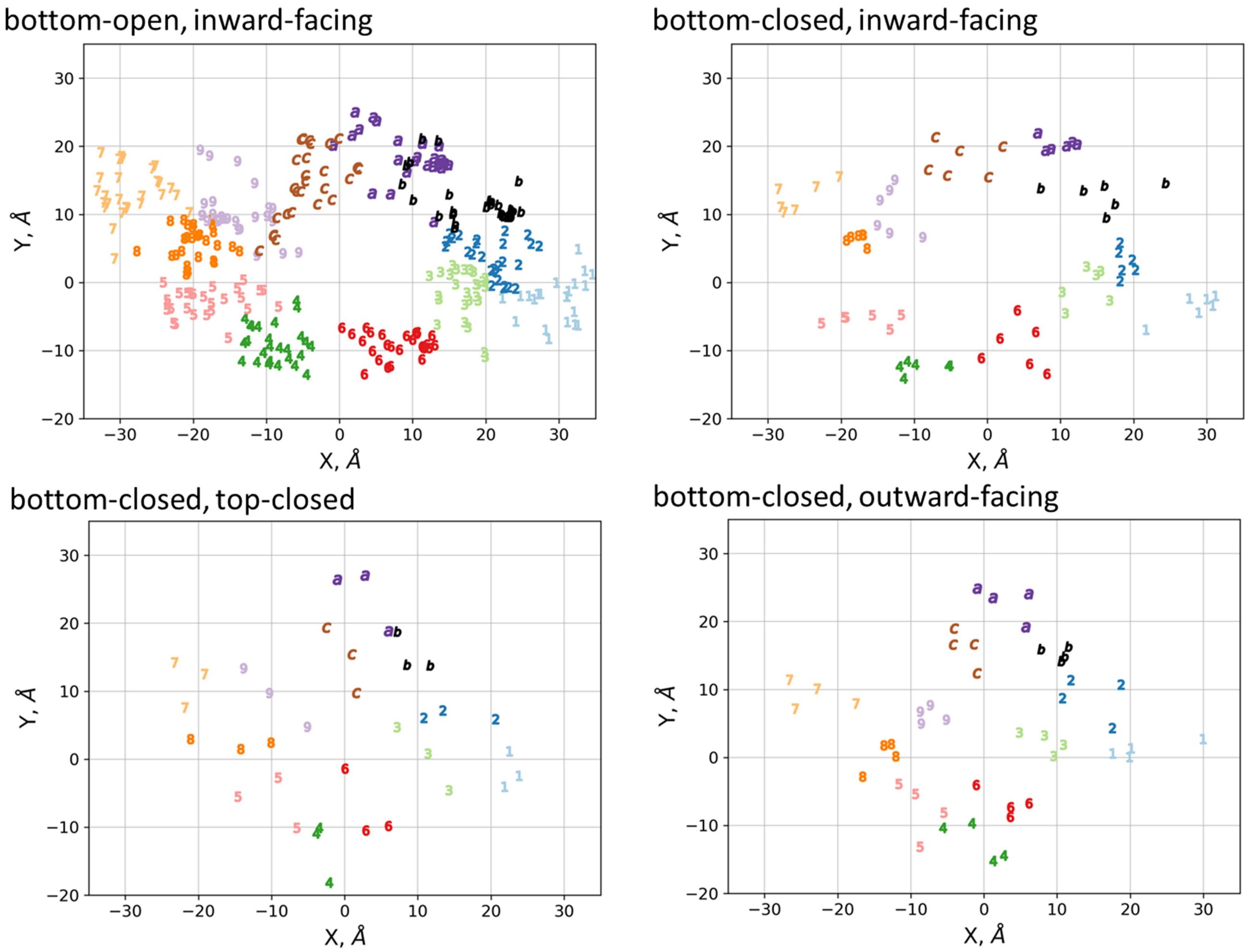

EXTRACELLULAR ENDS

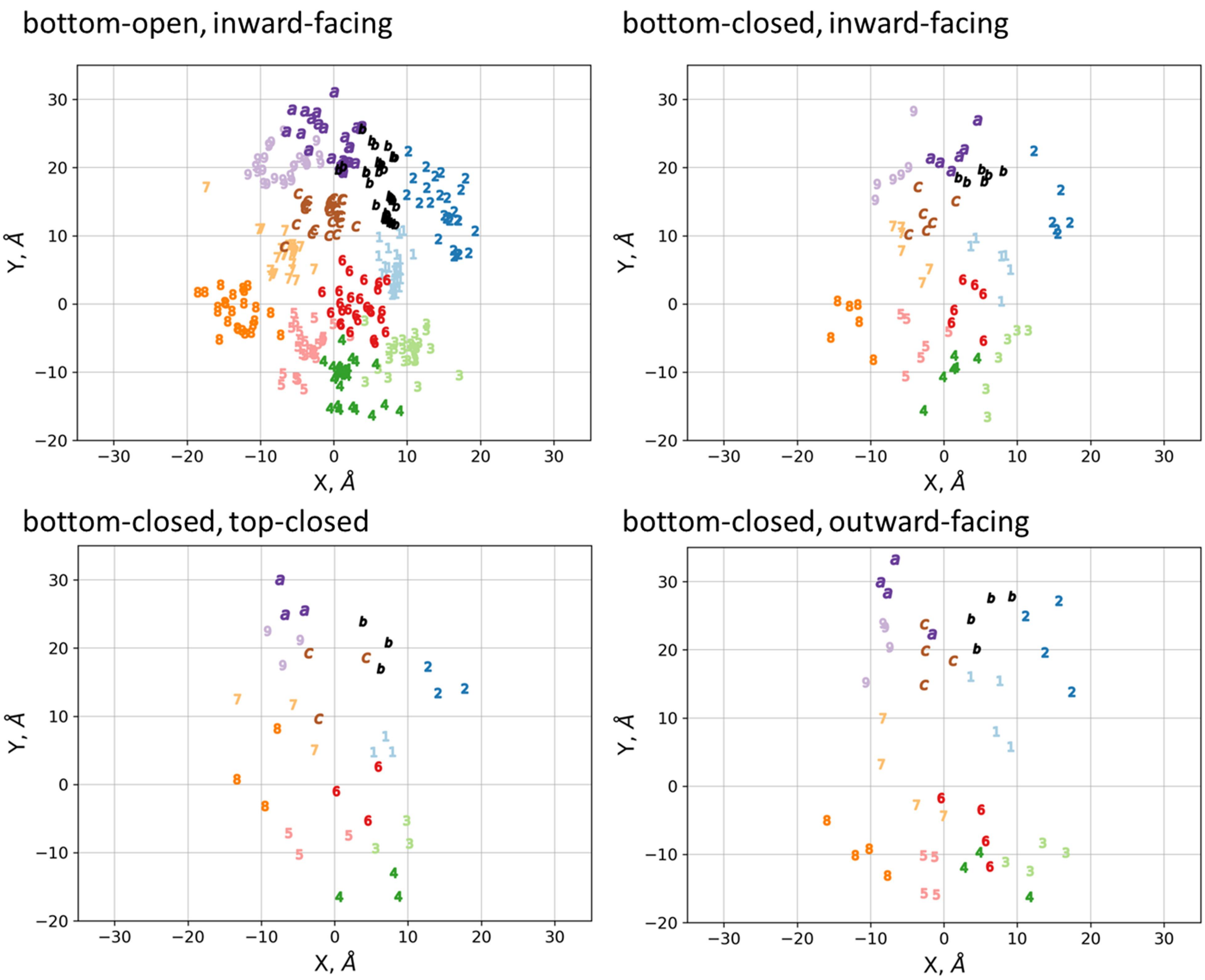

Figure S5. Comparing structures based on RMSD is a valid approach, but does not reveal the source of differences. Moreover, some structures were placed close to each other on the cluster tree, such as (3QF4 and 4AYX) and (2HYD and 4PL0), while they exhibit quite different conformations. 3QF4 (pale green) exhibits opened ICDs and closed NBDs. In contrast, the NBDs of 4AYX (blue) do not possess any contact. 2HYD (red) is widely open at the top, but 4PL0 (turquois) is closed to the extracellular space. Pairwise RMSD (root mean square deviation of atomic positions) values were calculated with PyMOL and used in hierarchical clustering (SciPy, linkage method: ward, Matplotlib for drawing a dendrogram).

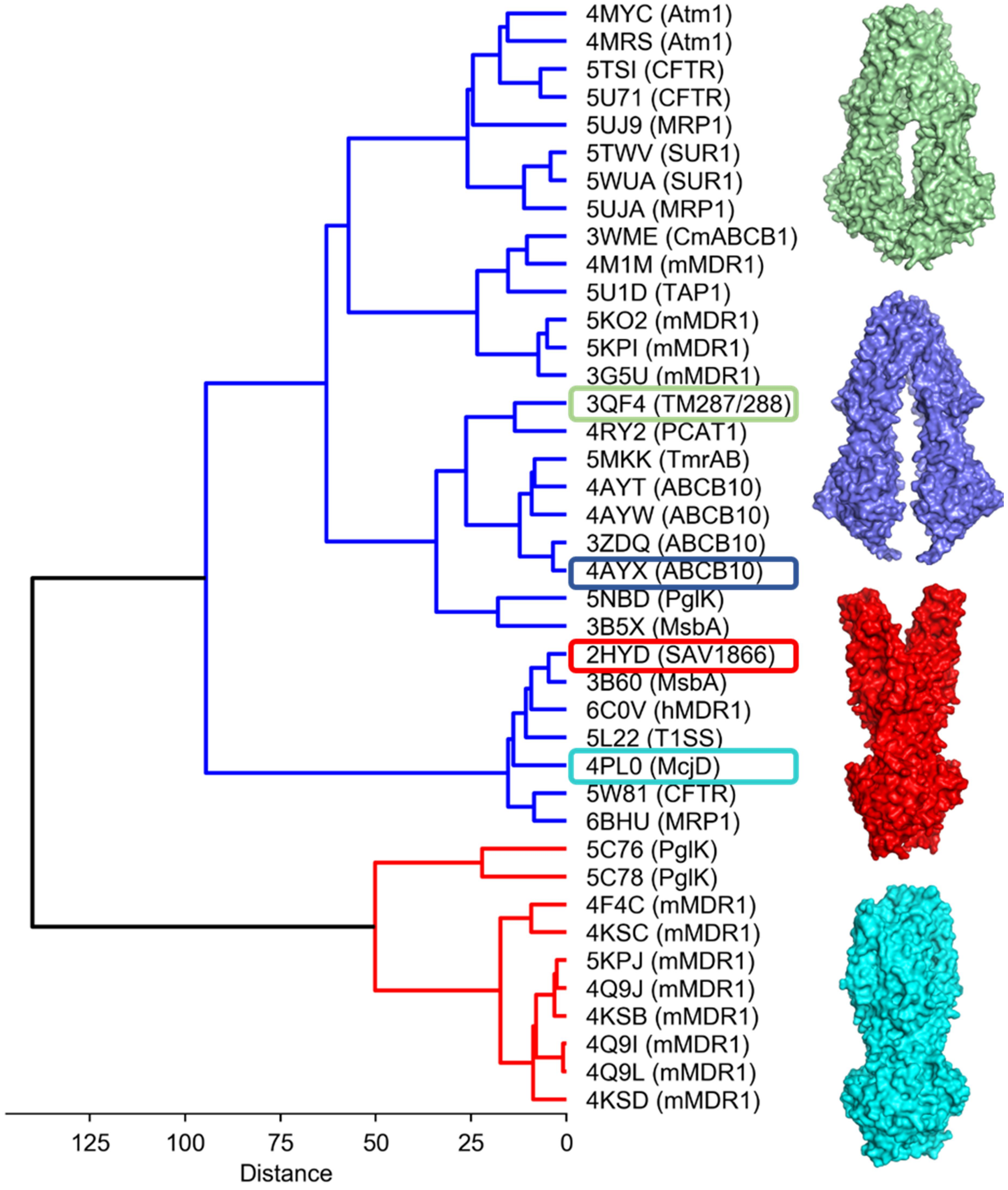

Figure S6. Selected vectors can reveal differences between conformations. The conftors can be projected to 2D for aiding visual inspection and comparison of structures. (**a**, **b**) The THX and THC conftors can visually differentiate “bottom-open, inward-facing” conformation (PDBID: 3G5U) from the “occluded” (PDBID: 4PL0) and “bottom-closed, outward-facing” (PDBID: 6C0V) conformations. Definition of THC conftors are shown in 3D for the “bottom-open, inward-facing” (**c**) and the “bottom-closed, outward-facing” (**d**) conformations. It is important to note that projecting 3D vectors onto a 2D pane results in some level of information loss.

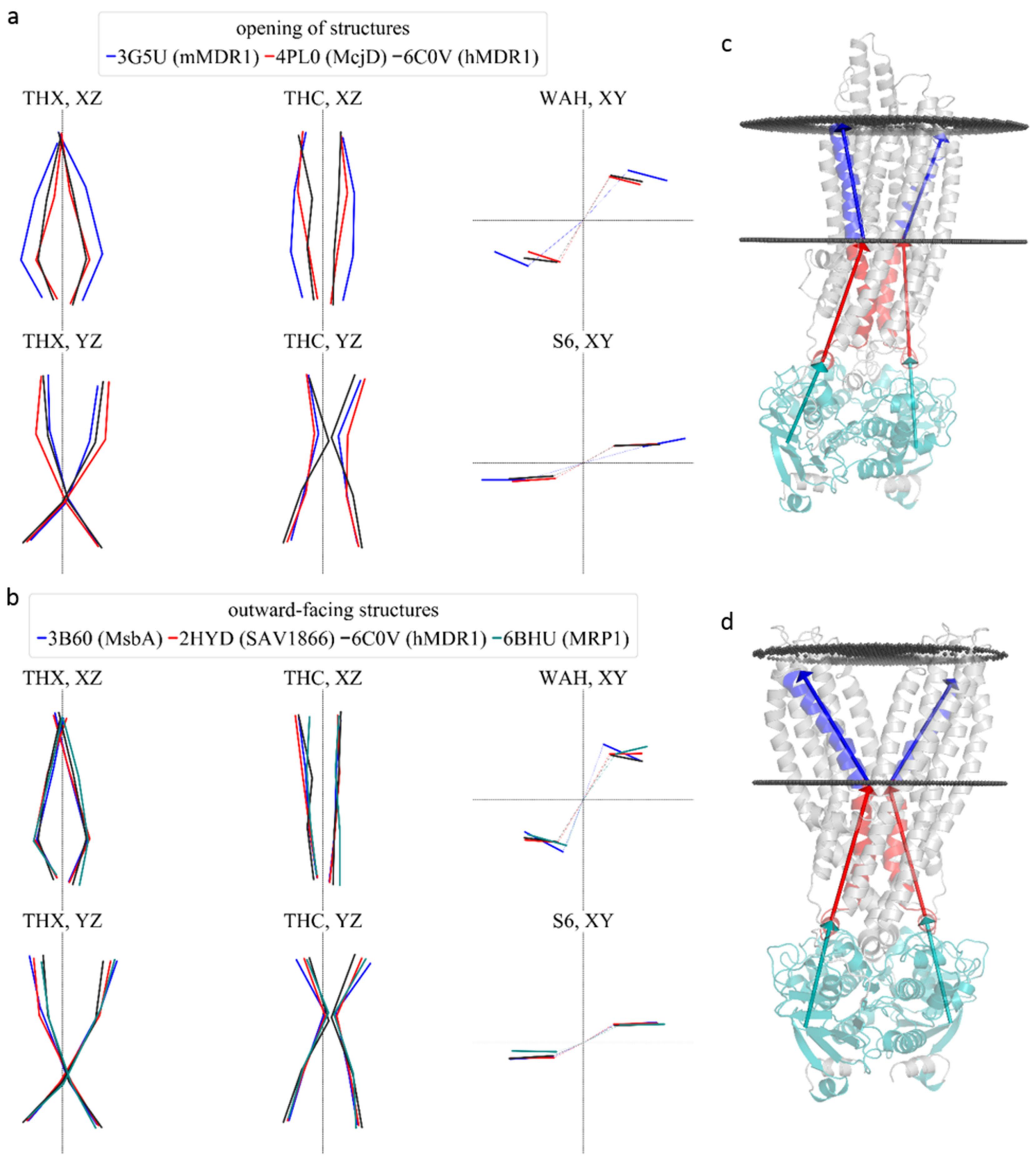

Figure S7. The intracellular positive blue ring is interrupted by a hydrophobic patch mediating protein-protein interaction. (**a**) A positively charged ring is formed by positively charged amino acids at the interface region of transmembrane helices, which is the manifestation of the positive-inside rule of TM proteins [7, 8]. APBS calculation was performed on ABCC1 (PDBID: 6BHU) and shown using PyMOL. (**b**) Similar calculation was performed after removal of the N-terminal L0/lasso region (green cartoon) preceding TH1 and exhibit a disruption of the positive ring by a hydrophobic patch (yellow circle). This hydrophobic surface area in the lipid head group layer indicates the binding location of a protein partner, which is the N-terminal region in this case. (**c**) If the N-terminal L0/lasso region is present then the patch is covered and a significant level of positive charge is observable (yellow circle), although it is not as intensive as on the other side of the protein (a). This is also true for other members of the ABCC subfamily [9] and indicates that surface electrostatics calculations may be used to predict protein-protein interaction sites in membrane proteins.

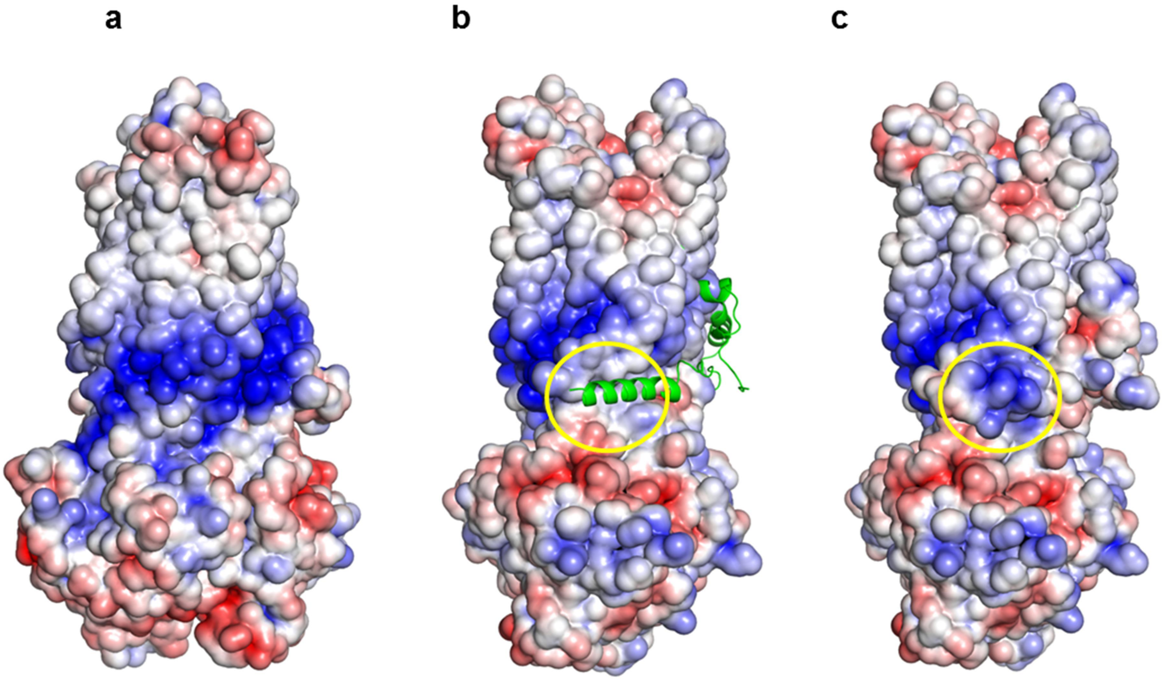

Table S1: ABC structures used in this study.

Name	PDBID	Conformation	Resolution	Method	Represented in OPM	Species
ABCB10	3ZDQ	bottom-open, inward-facing	2.85	X-ray diffraction	3ZDQ	*Homo sapiens*
ABCB10	4AYT	bottom-closed, inward-facing	2.85	X-ray diffraction	4AYT	*Homo sapiens*
ABCB10	4AYW	bottom-open, inward-facing	3.3	X-ray diffraction	4AYW	*Homo sapiens*
ABCB10	4AYX	bottom-open, inward-facing	2.9	X-ray diffraction	4AYX	*Homo sapiens*
Atm1	4MRN	bottom-open, inward-facing	2.35	X-ray diffraction	4MRS	*Novosphingobium aromaticivorans*
Atm1	4MRP	bottom-open, inward-facing	2.35	X-ray diffraction	4MRS	*Novosphingobium aromaticivorans*
Atm1	4MRR	bottom-open, inward-facing	2.35	X-ray diffraction	4MRS	*Novosphingobium aromaticivorans*
Atm1	4MRS	bottom-open, inward-facing	2.35	X-ray diffraction	4MRS	*Novosphingobium aromaticivorans*
Atm1	4MRV	bottom-open, inward-facing	2.35	X-ray diffraction	4MRS	*Novosphingobium aromaticivorans*
Atm1	4MYC	bottom-open, inward-facing	3.06	X-ray diffraction	4MYC	*Saccharomyces cerevisiae*
Atm1	4MYH	bottom-open, inward-facing	3.06	X-ray diffraction	4MYC	*Saccharomyces cerevisiae*
CFTR	5TSI	bottom-open, inward-facing	3.73	Electron microscopy	5TSI	*Danio rerio*
CFTR	5U71	bottom-open, inward-facing	3.87	Electron microscopy	5U71	*Homo sapiens*
CFTR	5UAK	bottom-open, inward-facing	3.87	Electron microscopy	5U71	*Homo sapiens*
CFTR	5W81	bottom-closed, top-closed	3.37	Electron microscopy	5W81	*Danio rerio*
CFTR model [10]	CFTR_ABCB10	bottom-closed, inward-facing	None	homology modeling	model	*Homo sapiens*
CFTR model	CFTR_IWF_DAS	bottom-open, inward-facing	None	modeling	model	*Homo sapiens*
CFTR model [10]	CFTR_MCJD	bottom-closed, top-closed	None	homology modeling	model	*Homo sapiens*
CFTR model	CFTR_MRP1	bottom-closed, outward-facing	None	modeling	model	*Danio rerio*
CFTR model	CFTR_OWF_DAS	bottom-closed, outward-facing	None	modeling	model	*Homo sapiens*
CFTR model	CFTR_SAV1866	bottom-closed, outward-facing	None	homology modeling	model	*Homo sapiens*
CFTR model [10]	CFTR_TM287288	bottom-closed, inward-facing	None	homology modeling	model	*Homo sapiens*
CmABCB1	3WME	bottom-closed, inward-facing	2.75	X-ray diffraction	3WME	*Cyanidoschyzon merolae*

CmABCB1	3WMF	bottom-closed, inward-facing	2.75	X-ray diffraction	3WME	*Cyanidoschyzon merolae*
hMDR1	6C0V	bottom-closed, outward-facing	3.4	Electron microscopy	6C0V	*Homo sapiens*
McjD	4PL0	bottom-closed, top-closed	2.7	X-ray diffraction	4PL0	*Escherichia coli*
McjD	5EG1	bottom-closed, top-closed	2.7	X-ray diffraction	4PL0	*Escherichia coli*
McjD	5OFR	bottom-closed, top-closed	2.7	X-ray diffraction	4PL0	*Escherichia coli*
mMDR1	3G5U	bottom-open, inward-facing	3.8	X-ray diffraction	3G5U	*Mus musculus*
mMDR1	3G60	bottom-open, inward-facing	3.8	X-ray diffraction	3G5U	*Mus musculus*
mMDR1	3G61	bottom-open, inward-facing	3.8	X-ray diffraction	3G5U	*Mus musculus*
mMDR1	4F4C	bottom-open, inward-facing	3.4	X-ray diffraction	4F4C	*Caenorhabditis elegans*
mMDR1	4KSB	bottom-open, inward-facing	3.8	X-ray diffraction	4KSB	*Mus musculus*
mMDR1	4KSC	bottom-open, inward-facing	4	X-ray diffraction	4KSC	*Mus musculus*
mMDR1	4KSD	bottom-open, inward-facing	4.1	X-ray diffraction	4KSD	*Mus musculus*
mMDR1	4M1M	bottom-open, inward-facing	3.8	X-ray diffraction	4M1M	*Mus musculus*
mMDR1	4M2S	bottom-open, inward-facing	3.8	X-ray diffraction	4M1M	*Mus musculus*
mMDR1	4M2T	bottom-open, inward-facing	3.8	X-ray diffraction	4M1M	*Mus musculus*
mMDR1	4Q9H	bottom-open, inward-facing	4	X-ray diffraction	4KSC	*Mus musculus*
mMDR1	4Q9I	bottom-open, inward-facing	3.78	X-ray diffraction	4Q9I	*Mus musculus*
mMDR1	4Q9J	bottom-open, inward-facing	3.6	X-ray diffraction	4Q9J	*Mus musculus*
mMDR1	4Q9K	bottom-open, inward-facing	4	X-ray diffraction	4KSC	*Mus musculus*
mMDR1	4Q9L	bottom-open, inward-facing	3.8	X-ray diffraction	4Q9L	*Mus musculus*
mMDR1	4XWK	bottom-open, inward-facing	3.8	X-ray diffraction	4Q9L	*Mus musculus*
mMDR1	5KO2	bottom-open, inward-facing	3.3	X-ray diffraction	5KO2	*Mus musculus*
mMDR1	5KOY	bottom-open, inward-facing	3.3	X-ray diffraction	5KO2	*Mus musculus*
mMDR1	5KPD	bottom-open, inward-facing	3.3	X-ray diffraction	5KO2	*Mus musculus*
mMDR1	5KPI	bottom-open, inward-facing	4.01	X-ray diffraction	5KPI	*Mus musculus*
mMDR1	5KPJ	bottom-open, inward-facing	3.5	X-ray diffraction	5KPJ	*Mus musculus*
MRP1	5UJ9	bottom-open, inward-facing	3.49	Electron microscopy	5UJ9	*Homo sapiens*

MRP1	5UJA	bottom-open, inward-facing	3.34	Electron microscopy	5UJA	*Homo sapiens*
MRP1	6BHU	bottom-closed, outward-facing	3.14	Electron microscopy	6BHU	*Homo sapiens*
MsbA	3B5X	bottom-closed, inward-facing	5.5	X-ray diffraction	3B5X	*Vibrio cholerae*
MsbA	3B60	bottom-closed, outward-facing	3.7	X-ray diffraction	3B60	*Vibrio cholerae*
PCAT1	4RY2	bottom-closed, inward-facing	3.61	X-ray diffraction	4RY2	*Clostridium thermocellum*
PglK	5C76	bottom-open, inward-facing	3.94	X-ray diffraction	5C76	*Campylobacter jejuni*
PglK	5C78	bottom-open, inward-facing	2.9	X-ray diffraction	5C78	*Campylobacter jejuni*
PglK	5NBD	bottom-open, inward-facing	3.9	X-ray diffraction	5NBD	*Campylobacter jejuni*
SAV1866	2HYD	bottom-closed, outward-facing	3	X-ray diffraction	2HYD	*Straphylococcus aureus*
SAV1866	2ONJ	bottom-closed, outward-facing	3	X-ray diffraction	2HYD	*Straphylococcus aureus*
SUR1	5TWV	bottom-open, inward-facing	6.3	Electron microscopy	5TWV	*Rattus norvegicus*
SUR1	5WUA	bottom-open, inward-facing	5.6	Electron microscopy	5WUA	*Mus musculus*
T1SS	5L22	bottom-closed, top-closed	3.15	X-ray diffraction	5L22	*Aquifex aeolicus*
TAP1	5U1D	bottom-open, inward-facing	3.97	Electron microscopy	5U1D	*Homo sapiens*
TM287/288	3QF4	bottom-closed, inward-facing	2.9	X-ray diffraction	3QF4	*Thermotoga maritima*
TM287/288	4Q4A	bottom-closed, inward-facing	2.9	X-ray diffraction	3QF4	*Thermotoga maritima*
TM287/288	4Q4H	bottom-closed, inward-facing	2.9	X-ray diffraction	3QF4	*Thermotoga maritima*
TM287/288	4Q4J	bottom-closed, inward-facing	2.9	X-ray diffraction	3QF4	*Thermotoga maritima*
TmrAB	5MKK	bottom-closed, inward-facing	2.7	X-ray diffraction	5MKK	*Thermus thermophilus*

Table S2: ABC conftors.

THX1	$\overrightarrow{COG(TH4_{int}, TH5_{int})COG(TH4_{ext}, TH5_{ext})}$
THX2	$\overrightarrow{COG(TH10_{int}, TH11_{int})COG(TH10_{ext}, TH11_{ext})}$
ICX1	$\overrightarrow{COG(CH2_{start}, CH2_{end})COG(TH4_{int}, TH5_{int})}$
ICX2	$\overrightarrow{COG(CH4_{start}, CH4_{end})COG(TH10_{int}, TH11_{int})}$
NBDX1	$\overrightarrow{COG(NBD2S8_{start}, NBD2S9_{end})COG(CH2_{start}, CH2_{end})}$
NBDX2	$\overrightarrow{COG(NBD1S8_{start}, NBD1S9_{end})COG(CH4_{start}, CH4_{end})}$
THV1	$\overrightarrow{TH2_{int}TH2_{ext}}$
THV2	$\overrightarrow{TH8_{int}TH8_{ext}}$
ICV1	$\overrightarrow{COG(CH1_{start}, CH1_{end})TH2_{int}}$
ICV2	$\overrightarrow{COG(CH3_{start}, CH3_{end})TH8_{int}}$
NBDV1	$\overrightarrow{COG(NBD1S8_{start}, NBD1S9_{end})COG(CH1_{start}, CH1_{end})}$
NBDV2	$\overrightarrow{COG(NBD2S8_{start}, NBD2S9_{end})COG(CH3_{start}, CH3_{end})}$
THC3	$\overrightarrow{TH3_{int}TH3_{ext}}$
THC9	$\overrightarrow{TH9_{int}TH9_{ext}}$
ICC3	$\overrightarrow{COG(CH1_{start}, CH1_{end})TH3_{int}}$
ICC9	$\overrightarrow{COG(CH3_{start}, CH3_{end})TH9_{int}}$
NBDC3	$\overrightarrow{COG(NBD1S8_{start}, NBD1S9_{end})COG(CH1_{start}, CH1_{end})}$
NBDC9	$\overrightarrow{COG(NBD2S8_{start}, NBD2S9_{end})COG(CH3_{start}, CH3_{end})}$
WAH1	$\overrightarrow{WAH1_{end}WAH1_{start}}$
WAH2	$\overrightarrow{WAH2_{end}WAH2_{start}}$
NBD1_S6	$\overrightarrow{NBD1S6_{start}NBD1S6_{end}}$
NBD2_S6	$\overrightarrow{NBD2S6_{start}NBD2S6_{end}}$
WAH1-SIG2	$\overrightarrow{WAH1_{start}SIG2_{start}}$
WAH2-SIG1	$\overrightarrow{WAH2_{start}SIG1_{start}}$

COG: center of geometry
ext: extracellular membrane plane
int: intracellular membrane plane

Table S3: Angles and lengths of conftors.

	bottom-open, inward-facing (n=27)	bottom-closed, inward-facing (n=6)	bottom-closed, top-closed (n=3)	bottom-closed, outward-facing (n=4)
	**Angle between conftors, deg**			
**TH1&TH3**	32.79 (±0.96)	39.78 (±1.45)	34.85 (±7.46)	50.92 (±4.61)
**TH7&TH9**	36.48 (±1.14)	34.53 (±1.66)	29.64 (±8.59)	48.33 (±5.21)
**THX1&THX2**	46.13 (±1.78)	39.73 (±2.35)	27.25 (±5.91)	35.04 (±2.38)
**ICX1&ICX2**	50.67 (±1.77)	44.58 (±2.17)	52.31 (±5.46)	54.02 (±2.81)
**NBDX1&NBDX2**	74.68 (±2.02)	75.36 (±4.87)	85.32 (±3.63)	82.84 (±0.72)
**THV1&THV2**	37.85 (±1.31)	36.76 (±2.91)	22.79 (±4.49)	40.35 (±4.49)
**ICV1&ICV2**	42.70 (±1.24)	37.96 (±3.32)	53.39 (±2.62)	60.12 (±2.01)
**NBDV1&NBDV2**	26.73 (±1.88)	27.39 (±4.56)	34.23 (±3.22)	27.74 (±2.57)
**WAH1&WAH2**	104.21 (±3.77)	123.09 (±5.20)	104.20 (±9.10)	110.49 (±3.59)
**NBD_S91&NBD_S92**	164.53 (±1.60)	172.13 (±2.60)	173.36 (±3.14)	174.83 (±1.17)
**THX1&ICX1**	13.64 (±1.05)	19.88 (±3.12)	18.85 (±5.16)	13.76 (±2.09)
**THX2&ICX2**	14.52 (±1.05)	16.30 (±2.91)	23.07 (±3.41)	15.65 (±3.32)
**ICX1&NBDX1**	37.68 (±1.55)	37.71 (±2.25)	28.23 (±7.81)	30.91 (±3.89)
**ICX2&NBDX2**	37.53 (±1.25)	40.41 (±1.86)	37.89 (±1.38)	34.82 (±3.06)
**THV1&ICV1**	18.88 (±1.59)	17.47 (±0.91)	21.14 (±7.78)	16.67 (±4.26)
**THV2&ICV2**	19.24 (±1.52)	20.70 (±1.28)	30.94 (±11.66)	15.63 (±2.91)
**ICV1&NBDV1**	14.77 (±0.99)	12.39 (±1.02)	13.78 (±3.30)	15.52 (±0.76)
**ICV2&NBDV2**	14.38 (±1.37)	13.36 (±1.58)	15.41 (±7.04)	17.60 (±2.45)
	**Distances between conftor ends, Å**			
**THX1&THX2_ext**	30.97 (±0.59)	29.68 (±1.76)	35.09 (±2.25)	40.63 (±3.12)
**ICX1&ICX2_ext**	35.39 (±0.68)	35.91 (±1.27)	33.36 (±2.93)	32.50 (±1.10)
**NBDX1&NBDX2_ext**	49.46 (±1.66)	38.93 (±1.66)	28.57 (±0.35)	28.70 (±0.95)
**NBDX1&NBDX2_int**	47.05 (±2.12)	27.84 (±4.09)	39.50 (±0.55)	40.04 (±1.38)
**THV1&THV2_ext**	32.64 (±0.67)	33.35 (±1.14)	28.23 (±2.26)	40.47 (±2.26)
**ICV1&ICV2_ext**	41.77 (±0.86)	37.49 (±0.85)	31.71 (±3.67)	29.69 (±1.49)
**NBDV1&NBDV2_ext**	43.96 (±1.40)	32.07 (±2.21)	28.30 (±2.23)	30.31 (±0.83)
**WAH1&WAH2_int**	59.70 (±1.64)	44.80 (±1.47)	40.83 (±0.90)	42.84 (±0.82)
**NBD_S91&NBD_S92_ext**	42.33 (±2.04)	29.50 (±2.07)	20.81 (±0.37)	20.75 (±0.05)

Table S4: Typical APBSmem parameters used in our study.

PDBID	3QF4*
force field	Parse
counter ions	1.0 0.15 0.95<br>-1.0 0.15 0.95
temperature	298.15 K
grid dimensions	161 161 161
coarse grid size	560 445 1155
medium grid size	224 178 462
fine grid size	112 89 231
protein dielectric	2
membrane dielectric	2
headgroup dielectric	80
solvent dielectric	80
membrane thickness	48
membrane bottom	-24.0
headgroup thickness	8.0 Å
upper/lower exclusion radii	-
grid center	origin
solution method	lpbe
boundary condition	zero
membrane potential	0 mV
charge model	spl2
surface model	mol
surface spline width	N/A
solvent probe radius	1.4 Å
surface sphere density	10 Å^{-2}

* Values shown for PDBID:3QF4, since box size and associated parameters depend on protein size.

References

1. Periole, X., et al., *Combining an Elastic Network With a Coarse-Grained Molecular Force Field: Structure, Dynamics, and Intermolecular Recognition.* J Chem Theory Comput, 2009. **5**(9): p. 2531-43.
2. Wassenaar, T.A., et al., *Computational Lipidomics with insane: A Versatile Tool for Generating Custom Membranes for Molecular Simulations.* J Chem Theory Comput, 2015. **11**(5): p. 2144-55.
3. Qi, Y., et al., *CHARMM-GUI Martini Maker for Coarse-Grained Simulations with the Martini Force Field.* J Chem Theory Comput, 2015. **11**(9): p. 4486-94.
4. de Jong, D.H., et al., *Martini straight: Boosting performance using a shorter cutoff and GPUs.* Computer Physics Communications, 2016. **199**: p. 1-7.
5. Jo, S., et al., *CHARMM-GUI: a web-based graphical user interface for CHARMM.* J Comput Chem, 2008. **29**(11): p. 1859-65.
6. Morgan, J.L.W., J.F. Acheson, and J. Zimmer, *Structure of a Type-1 Secretion System ABC Transporter.* Structure, 2017. **25**(3): p. 522-529.
7. Elazar, A., et al., *Interplay between hydrophobicity and the positive-inside rule in determining membrane-protein topology.* Proc Natl Acad Sci U S A, 2016. **113**(37): p. 10340-5.
8. von Heijne, G., *Control of topology and mode of assembly of a polytopic membrane protein by positively charged residues.* Nature, 1989. **341**(6241): p. 456-8.
9. Tordai, H., I. Leveles, and T. Hegedus, *Molecular dynamics of the cryo-EM CFTR structure.* Biochem Biophys Res Commun, 2017. **491**(4): p. 986-993.
10. Corradi, V., P. Vergani, and D.P. Tieleman, *Cystic Fibrosis Transmembrane Conductance Regulator (CFTR): CLOSED AND OPEN STATE CHANNEL MODELS.* J Biol Chem, 2015. **290**(38): p. 22891-906.
